# FUBP3 enhances HIV-1 transcriptional activity and regulates immune response pathways in T cells

**DOI:** 10.1016/j.omtn.2025.102525

**Published:** 2025-03-25

**Authors:** Quentin M.R. Gibaut, Chuan Li, Anqi Cheng, Ines Moranguinho, Luisa P. Mori, Susana T. Valente

**Affiliations:** 1 The Skaggs Graduate School of Chemical and Biological Sciences, The Scripps Research Institute, Jupiter, FL 33458, USA; 2 Department of Immunology and Microbiology, The Herbert Wertheim UF Scripps Institute for Biomedical Innovation & Technology, Jupiter, FL 33458, USA

**Keywords:** MT: Oligonucleotides, Therapies and Applications, FUBP3, HIV, transcription, TAR, RNA stability, Tat, T cells, immune response

## Abstract

Far-upstream element-binding protein 3 (FUBP3) was identified at actively transcribing HIV promoters through chromatin affinity purification and mass spectrometry. Known for regulating cellular processes such as transcription and translation by binding to DNAs and RNAs, FUBP3’s role in HIV transcriptional regulation was previously unrecognized. This study reveals that FUBP3 enhances HIV-1 transcriptional activation by interacting with Tat and *trans*-activation response (*TAR*)-RNA, critical for boosting viral transcription through recruitment of activating factors that promote RNA polymerase II (RNAPII) elongation. Transcriptomic analysis, chromatin immunoprecipitation, and biochemical assays demonstrated that FUBP3 associates with and stabilizes *TAR*-RNA, in a Tat-dependent manner, and enhances Tat steady-state levels via interaction with Tat’s basic domain. Suppressing FUBP3 decreased HIV-1 transcription and altered expression of host genes linked to T cell activation and inflammation, underscoring its broad regulatory impact. Additionally, FUBP3 was enriched at active promoters, confirming its role in transcriptional regulation at specific genomic locations. These findings highlight FUBP3’s critical role in the HIV-1 life cycle and suggest its potential as a therapeutic target in HIV-1 infection. Additionally, this study expands our understanding of FUBP3’s functions in oncogenic and inflammatory pathways.

## Graphical abstract

**Figure:**
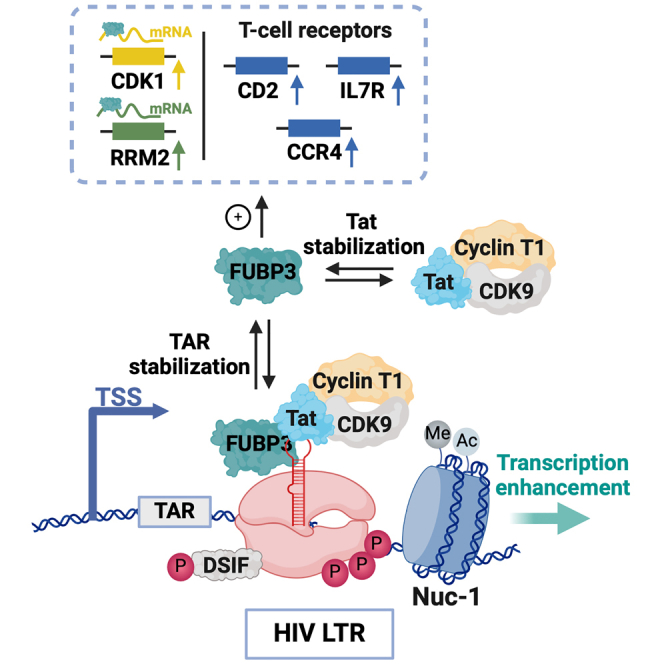

## Teaser

Valente and colleagues identified FUBP3 as a critical host factor for HIV-1 transcription, enhancing Tat-TAR interactions and RNAPII elongation. This study uncovers FUBP3’s dual role in viral activation and immune regulation, providing insights into host-virus dynamics and highlighting its potential as a therapeutic target in HIV-1 infection and related pathologies.

## Introduction

Despite the life-saving benefits of antiretroviral therapy (ART), HIV-1 infection remains incurable due to the persistence of latently infected CD4^+^T cells, which reactivate upon ART interruption.^1^^,^^2^^,^^3^^,^^4^^,^^5^ The HIV-1 reservoir is established early after infection,^6^^,^^7^ and its persistence is facilitated by complex mechanisms that regulate transcriptional silencing and activation. Specifically, the viral transactivator protein Tat, along with various cellular transcription factors (TFs) and chromatin regulatory factors (CRFs),^8^^,^^9^^,^^10^^,^^11^ contribute to the intricate interplay between these two states. Identifying and characterizing the factors that modulate HIV-1 transcription is essential for developing effective strategies aimed at achieving viral eradication.

The HIV-1 promoter, also called the 5′ long terminal repeat (LTR) exhibits a well-defined nucleosome (Nuc) organization, with Nuc-0 and Nuc-1 flanking the transcription start site (TSS).^12^^,^^13^^,^^14^ It includes binding sites for host TFs, such as TATA binding protein; general TFs such as TFIIA, TFIIB, and TFIIF; nuclear factor kappa B (NF-κB); and specificity protein 1 (Sp1), which initiate the formation of the preinitiation complex (PIC).^15^^,^^16^^,^^17^^,^^18^ Following the recruitment of TFIIH, which includes CDK7, RNA polymerase II (RNAPII) is phosphorylated at the Ser5 residue of the carboxy-terminal domain (RNAPII-CTD) by CDK7.^19^ The first transcript to be generated is the *trans*-activation response (*TAR*) RNA, which is ∼59 nt long following the TSS and folds into a dynamic hairpin secondary structure.^20^^,^^21^

The viral transactivator protein Tat, initially produced from splicing of rare full-length transcripts, facilitates the recruitment of the transcriptional elongation factor b (P-TEFb), composed of CDK9 and cyclin T1 (CCNT1), to the *TAR*-RNA. This dynamic interaction results in the exponential enhancement of RNAPII elongation and HIV-1 gene expression, mediated by phosphorylation events such as at the Ser2 of RNAPII-CTD, which promotes RNAPII pause release. Stabilization of *TAR*-RNA is critical for maintaining its structural integrity, ensuring effective interactions with Tat and its recruited cofactors.^21^^,^^22^^,^^23^ Disruption of *TAR*-RNA stability or its interaction with Tat can impair transcriptional activation and compromise efficient viral gene expression, as demonstrated by the effects of Tat/*TAR* inhibitors.^24^^,^^25^^,^^26^^,^^27^ Furthermore, Tat recruits the super elongation complex (SEC), histone acetylases, and chromatin remodelers to the transcriptional machinery, thereby enabling efficient transcriptional amplification.^28^^,^^29^^,^^30^

The initiation of HIV-1 transcription, mediated by PIC formation and RNAPII recruitment, represents a critical step leading to the initial burst of viral mRNA production.^11^ This phase enables the generation of Tat and the establishment of the Tat-*TAR* feedback loop.^13^^,^^14^^,^^31^^,^^32^^,^^33^^,^^34^ Despite significant progress in understanding HIV-1 transcriptional regulation, many TFs involved in this process remain unknown. Elucidating the mechanisms governing transcription from integrated HIV-1 proviruses and identifying host factors involved in HIV transcription may offer new avenues for curative strategies.

To determine enrichment or depletion of proteins in transcriptionally active or silenced HIV promoter loci, we previously reported on a selective and unbiased method for specific chromatin affinity immunoprecipitation (ChIP).^35^ This technique uses dCas9/gRNA-mediated targeting, coupled with mass spectrometry (ChAP-MS), to achieve precise enrichment and protein identification.^35^ This approach revealed proteins participating in HIV-1 transcriptional regulation, including the chaperone protein p32, which was found to enhance HIV transcription by supporting Tat-*TAR*/P-TEFb/RNAPII interaction.^35^ The far-upstream element-binding protein 3 (FUBP3) was also identified among the most enriched factors bound to the active HIV-1 promoter, and the significance of these findings is explored here.

FUBP3 is an intriguing protein known to interact with single-stranded DNA within a specific regulatory DNA sequence called the far-upstream sequence element (FUSE), particularly associated with the *c-Myc* gene to modulate gene expression.^36^^,^^37^ While initially characterized in the context of the *c-Myc* proto-oncogene regulation, similar regulatory elements may exist for other genes.^38^^,^^39^^,^^40^^,^^41^ FUBP3, along with FUBP1 and FUBP2, constitute an ancient protein family that bind to FUSE and control *c-Myc* proto-oncogene transcription through interactions with TFIIH.^42^^,^^43^^,^^44^ FUBP3 also binds long non-coding RNAs (lncRNAs) such as lnc-*CMPK2*, which regulates *c-Myc* expression^45^; lnc-*GATA2-AS1*, which represses the transcription of tumor-suppressive genes^46^; lnc-*TRMP-S*, which stalls the translation of p53^47^; and lnc-*EST12*, which modulates the host immune response during *Mycobacterium tuberculosis* infection.^48^ In the realm of virology, FUBP3 has been found to regulate the replication of enterovirus 71 through interactions with specific RNA regions.^49^ Notably, FUBP3 is upregulated in CD4^+^T cells of individuals with viremic HIV infection, although its functional significance has not been explored.^50^ Present in both nuclear and cytoplasmic compartments, FUBP3 exhibits context-dependent modulation of gene expression.^44^^,^^51^

Here, we aimed to build on our previous findings and elucidate the mechanism by which FUBP3 regulates HIV-1 transcription. Our findings reveal that FUBP3 positively regulates HIV-1 transcription and infection in various cell lines and primary CD4^+^T cells. Our investigation determined that FUBP3 is required for RNAPII processing through the HIV genome, and it operates in a Tat-dependent manner. Specifically, FUBP3 C-terminal binds Tat’s basic domain, while FUBP3 N- and C-terminal regions are involved in its binding to the *TAR*-RNA. Notably, FUBP3 stabilizes both Tat protein steady state and *TAR*-RNA half-life, enhancing RNAPII transcriptional elongation. Furthermore, FUBP3 interacts with a set of actively transcribing promoters located upstream of the TSS, underscoring its involvement in transcriptional regulation at specific genomic loci. Beyond its role in HIV, FUBP3 modulates the expression of genes linked to T cell activation, inflammation, and oncogenic pathways while also recognizing numerous mRNAs associated with viral infection, cell cycle regulation, and cancer pathways. Our findings collectively suggest that FUBP3 is a multifaceted protein that influences various cellular processes and importantly serves as a positive host regulator of HIV-1 transcription.

## Results

### FUBP3 identified by ChAP-MS is required for acute HIV-1 infection

We previously reported on host factors directly associating with active and latent HIV-1 promoters identified through a selective and unbiased approach of chromatin immunoprecipitation (ChIP) (using a dCas9 and gRNAs) followed by mass spectrometry and dubbed ChAP-MS.^35^^,^^52^ In brief, ChAP-MS was performed using the M1 single-cell clone derived from HeLa-CD4 cells, transfected with a catalytically inactive dCas9 (dCas9) tagged with protein A and a mixture of four gRNAs targeting the HIV promoter, as well as a negative-control gRNA targeting GFP. Cells expressing dCas9 fused to protein A and gRNAs were crosslinked, chromatin was fragmented, and the HIV promoter was specifically enriched using rabbit immunoglobulin (Ig)G to pull down dCas9-ProtA. The isolated proteins were then separated by SDS-PAGE, followed by gel excision, in-gel trypsin digestion, and mass spectrometry analysis (liquid chromatography-tandem mass spectrometry [LC-MS/MS]). M1 cells treated solely with ART released an average of 1,000 pg/mL of capsid p24 into the supernatant and were considered chronically infected and actively transcribing. To investigate the latent configuration of the HIV-1 promoter, cells were treated with the Tat inhibitor didehydro-Cortistatin A (dCA).^53^^,^^54^^,^^55^ This treatment lowered viral production to below the detection limit within 24 days and inhibited viral reactivation in response to various latency-reversing agents applied on day 44, including tumor necrosis factor (TNF)-α, Trichostatin A (TSA), suberoylanilide hydroxamic acid (SAHA), and phorbol myristate acetate (PMA)^35^ (more experimental details can be found in Li et al.^35^). This ChAP-MS study revealed top protein candidates enriched in ART-treated (active LTR) and ART+dCA-treated (latent LTR) M1 cells. These were ranked based on peptide enrichment revealing several known proteins typically associated with active transcription such as AIMP1,^56^^,^^57^ PABPC1,^58^^,^^59^ RUVBL1,^60^ RPA1,^61^ and XRCC6,^62^^,^^63^ as well as proteins expected to be found in silenced promoters such as TRIM28,^64^ H1, H2, and H4 histones.^65^^,^^66^^,^^67^ The minimal size of the HIV LTR promoter, coupled with detection constrains of MS, may explain the absence of detection of certain known HIV promoter-associated factors in our study. FUBP3 was identified as highly enriched in actively transcribing HIV promoters, yet there was limited knowledge about its cellular function. Additionally, there had been no prior association reported between FUBP3 and HIV transcription (Figure S1).

**Figure 1:**
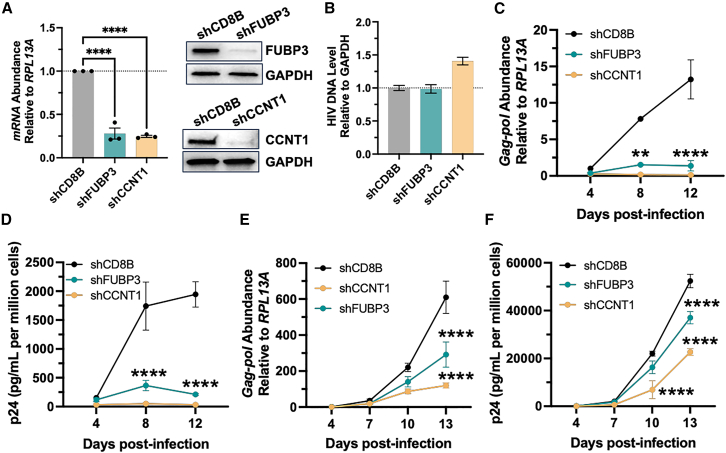
FUBP3 is important for HIV transcription and replication (A) Knockdown efficiency for FUBP3 and CCNT1 monitored by RT-qPCR (left) and western blot (right) in Jurkat CD4^+^T cells. (B) HIV integration in Jurkat CD4^+^T cells quantified by ALU-PCR assay. (C) Effect of FUBP3 and CCNT1 depletion on *Gag-pol* abundance in infected Jurkat CD4^+^T cells over time post infection. (D) Viral protein abundance determined by ELISA in infected Jurkat CD4^+^T cell medium. (E) Effect of FUBP3 and CCNT1 depletion on *Gag-pol* abundance in infected primary CD4^+^T cells over time post infection. (F) Viral protein abundance determined by ELISA in infected primary CD4^+^T cell medium. ∗∗*p* < 0.01, ∗∗∗∗*p* < 0.0001, as determined by two-way ANOVA with multiple comparisons. All data are reported as the mean ± SEM.

To investigate the role of FUBP3 in HIV replication and transcription, we performed depletion experiments using retroviral vectors (pMKO-puro) expressing short hairpin RNAs (shRNAs) targeting *FUBP3* (shFUBP3), *CCNT1* (shCCNT1), and *CD8B* (shCD8B). These shRNAs were packaged into virus-like particles (VLPs), transduced into Jurkat cells, and puromycin selected (2 μg/mL) prior to infection with the HIV NL4-3 isolate. Cells were lysed for RNA and protein analysis over time at 4, 8, and 12 days post infection (dpi). The targeted genes *FUBP3* and *CCNT1* were successfully depleted, as determined by western blot and RT-qPCR, with ∼70% reduction in mRNA compared to shCD8B used as a non-targeting control (Figure 1A). HIV provirus integration was also monitored by Alu-qPCR and showed relative uniform integration across conditions (Figure 1B), which rules out any influence of FUBP3 on viral integration. Notably, depletion of FUBP3 led to an approximate 80% reduction in viral mRNA production at 8 and 12 dpi, a decrease similar to that observed with the depletion of CCNT1, a factor critical for transcriptional elongation (Figures 1C and S2A–S2C). This occurred without affecting cellular viability, as compared to shCD8B control (Figure S2D). Viral capsid p24 protein production was evaluated in the supernatant using an ELISA assay, confirming that depleting FUBP3 resulted in a decrease in viral production, comparable to the reduction observed with CCNT1 knockdown (Figure 1D).

A similar validation study of the role of FUBP3 was conducted in primary CD4^+^T cells isolated from three healthy donors. As before, depletion of FUBP3 and CCNT1 was confirmed by western blot and RT-qPCR, showing a reduction in target gene expression of ∼60% (Figure S3A). Upon infection with NL4-3, the HIV provirus was found homogeneously integrated among donors and conditions tested, as determined by qPCR (Figure S3B). Again, a decrease in viral mRNA and p24 production was observed throughout the 13 dpi with FUBP3 depletion, with ∼50% reduction in HIV mRNA and ∼30% reduction in p24 levels with effects accumulating over time (Figures 1E, 1F, and S3C). No significant changes in cell viability were observed as compared to shCD8B control (Figure S3D). Collectively, these findings suggest an important function for FUBP3 during HIV-1 replication, most likely during HIV transcription.

### FUBP3 is an HIV-1 transcriptional activator

**Figure 2:**
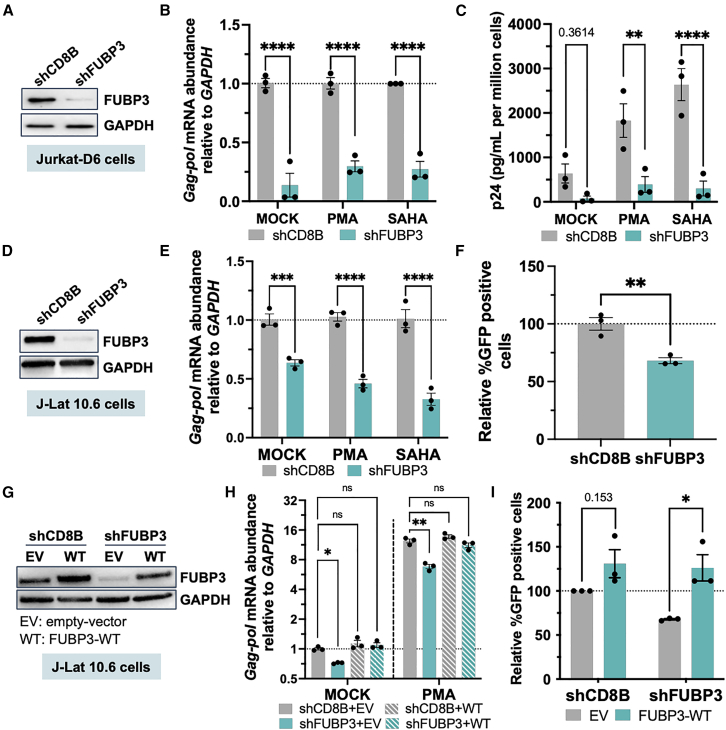
FUBP3 acts as an HIV transcription activator (A) shRNA depletion of FUBP3 and control CD8B in Jurkat-D6 cells by western blot. (B) *Gag-pol* transcript abundance upon FUBP3 depletion with or without stimulation (mock, PMA, SAHA) in Jurkat-D6 cells as determined by RT-qPCR. (C) Viral protein abundance determined by ELISA in Jurkat-D6 cell medium. (D) As for (A) but in J-Lat 10.6 cells. (E) *Gag-pol* transcript abundance upon FUBP3 depletion with or without stimulation (mock, PMA, SAHA) in J-Lat 10.6 cells. (F) Quantification of HIV (%GFP) upon stimulation with PMA. (G) Representative western blot analysis of FUBP3 and GAPDH protein levels in J-Lat 10.6 where FUBP3/CD8B were first depleted before the re-introduction of either FUBP3-WT (WT) or empty-vector (EV) clones. (H) *Gag-pol* transcript abundance with or without stimulation (mock or PMA) as determined by RT-qPCR. (I) Quantification of HIV (%GFP) upon stimulation with PMA. ∗*p* < 0.05, ∗∗*p* < 0.01, ∗∗∗*p* < 0.001, ∗∗∗∗*p* < 0.0001, as determined by two-way ANOVA with multiple comparisons. All data are reported as the mean ± SEM.

To rule out cell- or integration-dependent effects, we tested FUBP3’s ability to modulate HIV-1 transcription in additional human cell lines infected with HIV. First, we employed shRNAs to deplete FUBP3 in Jurkat-D6 cells (Figure 2A). These cells, a latently infected Jurkat cell clone (made and characterized in house), harbor a single integrated copy of HIV NL4-3 on chromosome 15 at position 72355395. Depletion of FUBP3 significantly reduced HIV transcription, both residual and activated by PMA (10 nM, 6 h) or SAHA (2 μM, 6 h) (Figures 2B and S4A). Transcripts including *TAR* and *Gag-pol* were assessed by RT-qPCR with specific primers, suggesting a general loss in transcription (Figures 2B and S4A). Additionally, a substantial decrease in p24 viral protein production was observed under FUBP3 knockdown conditions (Figure 2C). Of note, FUBP3 does not regulate FUBP1 and FUBP2, and vice versa, as no changes in mRNA levels for these factors was observed upon their respective depletion in Jurkat-D6 cells (Figures S4B–S4D).

Subsequently, we carried out shRNA-mediated depletion of FUBP3 in J-Lat 10.6 cells (Figures 2D and S5A). This latently infected cell line contains a full-length HIV-1 genome characterized by a non-functional Env due to a frameshift and Nef protein replaced by GFP (CVCL_8281) (PMID: 12682019). Similar to the findings in Jurkat-D6 cells, depletion of FUBP3 reduced both basal transcription and transcription activated by PMA (10 nM, 6 h) or SAHA (2 μM, 6 h). A statistically significant reduction in the levels of *TAR* and *Gag-pol* transcripts was observed via RT-qPCR (Figures 2E and S5B). Although the reduction following FUBP3 depletion is less pronounced in J-Lat 10.6 cells than in Jurkat-D6 cells, this difference is likely attributable to the intrinsic differences between these two latency models. Specifically, Jurkat-D6 cells typically display higher levels of basal HIV transcription than J-Lat 10.6 cells, even without stimulation. Flow cytometry analysis of GFP expression, which is indicative of HIV transcriptional activation, revealed a reduction in GFP-positive cells in FUBP3 depleted cells, confirming the loss in HIV transcription (Figures 2F and S5C). Finally, we reintroduced and overexpressed (OE) FUBP3 in J-Lat 10.6 cells from which FUBP3 had previously been depleted (Figures 2G and S5D). The overexpression of FUBP3 effectively improved or fully restored both basal and PMA (10 nM, 6 h) activated HIV *TAR*, and *Gag-pol* transcripts, as confirmed by RT-qPCR (Figures 2H and S5E). Additionally, the number of GFP-positive cells increased, as demonstrated by flow cytometry (Figures 2I and S5F). Collectively, these findings highlight the critical role of FUBP3 in HIV transcriptional regulation.

### FUBP3 regulates pathways associated with inflammation and HIV infection

**Figure 3:**
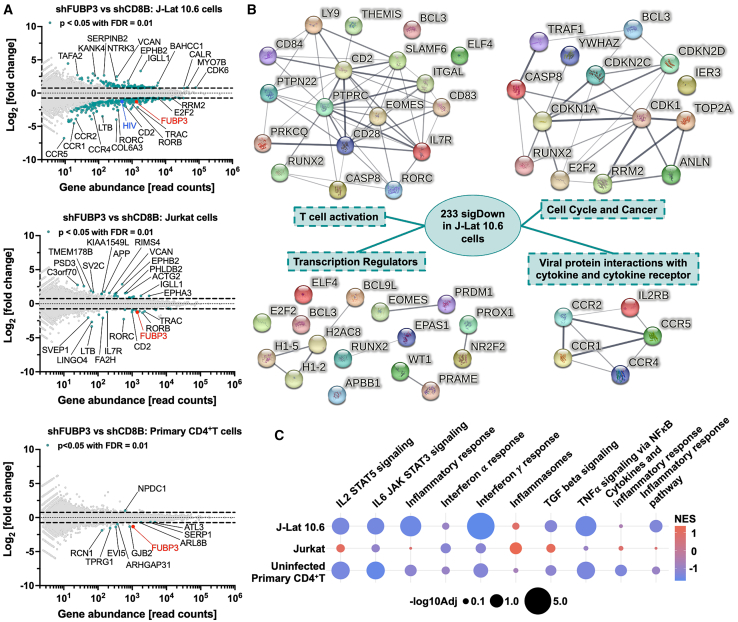
FUBP3 regulates pathways involved with inflammation and HIV infection (A) RNA-seq analysis of differentially expressed genes (DEGs) upon FUBP3 knockdown (KD) in J-Lat 10.6 cells (left), uninfected Jurkat cells (middle), and uninfected primary CD4^+^T cells (right). (B) Protein network of downregulated genes in J-Lat 10.6 cells upon FUBP3 depletion grouped by function using STRING db v12. Each node represents a protein, and line thickness indicates confidence in both functional and physical protein associations. (C) Bubble plot of a GSEA of the indicated pathways across the three cell models upon FUBP3 depletion. NES, normalized enrichment score.

To thoroughly explore the biological role of FUBP3, we performed transcriptome-wide analysis using total RNA collected from FUBP3 or CD8B depleted uninfected Jurkat, unstimulated J-Lat 10.6, and uninfected primary CD4^+^T cells. As anticipated, a significant reduction in FUBP3 expression was observed in FUBP3 depleted cells, as confirmed by the read count (transcripts per million [TPM]), RT-qPCR, and western blot compared to CD8B-knockdown controls while maintaining high cellular viability (Figures S6A and S6B). Additionally, in line with previous RT-qPCR data, a significant decrease in HIV expression was noted in J-Lat 10.6 cells where FUBP3 was depleted, further supporting FUBP3’s role as a positive regulator of HIV (Figure S7A). Following FUBP3 depletion, the transcriptome-wide study showed significant dysregulation of 352 genes in J-Lat 10.6 cells (233 downregulated and 121 upregulated), 44 genes in Jurkat cells (20 downregulated and 24 upregulated), and 10 genes in non-infected primary CD4^+^T cells (nine downregulated and one upregulated) (Figures 3A and S7B). The primary overlap among dysregulated genes was observed between J-Lat 10.6 and Jurkat cells, representing over 50% of DEGs in Jurkat cells. These two cellular models are more comparable to each other than to primary cells (Figure S7C). Interestingly, most genes dysregulated by shFUBP3 were found in J-Lat 10.6 cells and were predominantly downregulated rather than upregulated. This suggests that FUBP3 generally promotes gene expression across the transcriptome, particularly in the context of HIV-1 infection. Notably, the limited dysregulation of genes in primary CD4^+^T cells, which could be attributed to variability in gene expression among donors, suggest that therapeutically targeting FUBP3 may be relatively safe and hold significant therapeutic potential.

Among the genes downregulated by FUBP3 depletion, the nuclear receptors retinoic-acid-receptor-related orphan receptor β (*RORB*) and γ (*RORC*) were significantly downregulated in J-Lat 10.6 and Jurkat cells. At the protein level, RORC was also downregulated (Figure S8, RORB not tested). Although these receptors are primarily expressed in the central nervous system and may not relate to HIV biogenesis, their downmodulation could unveil new mechanistic pathways influenced by FUBP3.^68^^,^^69^ HIV-1 transcription is intricately linked to the activation state of the host cell and can be influenced by various cellular pathways. As both a DNA- and RNA-binding protein, FUBP3 is poised to regulate genes in T cells that are involved in T cell activation, with potential to indirectly affect HIV-1 dynamics. To further explore this, the upregulated and downregulated genes in J-Lat 10.6 were analyzed using the STRING database to illustrate their function and protein-protein interactions. Analysis of the upregulated gene list revealed clusters associated with cancer, cell migration, and nervous system development (Figure S9). In contrast, the analysis of the downregulated gene list highlighted pathways of potential relevance to HIV transcription and infection (Figure 3B). Notably, FUBP3 was found to regulate proteins involved with T cell activation, viral protein interaction with cytokine and cytokine receptors, and cell cycle regulation (Figure 3B). Additionally, several transcription and chromatin regulators were also significantly downregulated upon FUBP3 depletion that could have an influence on HIV transcription activation (Figure 3B). Further investigation is warranted to explore their specific contributions. This suggests new mechanistic pathways where FUBP3 regulates the expression of numerous T cell receptors, potentially influencing T cell activation, as shown by genes such as *CCR4* and *IL7R*, which were validated by RT-qPCR across the three cell lines (Figure S10). Additionally, the depletion of FUBP3 results in the downregulation of ribonucleotide reductase *RRM2*. RRM2 is crucial for maintaining dNTP pools for DNA biosynthesis, repair, and replication and it plays a critical role in cancer biology (Figure S10).^70^ Moreover, numerous genes downregulated by FUBP3 depletion are directly involved with HIV transcription and/or infection. These include *CD2*, *CD28*, *CD38*, *CD69*, *CCR1*, *CCR2*, *CCR5*, *ITGAL*, and *CDK1*.^71^^,^^72^^,^^73^^,^^74^^,^^75^^,^^76^^,^^77^^,^^78^ To ensure the shRNA selection method (e.g., puromycin) did not indirectly influenced the transcriptomic analysis outcome, we repeated the FUBP3 depletion studies using an alternative retroviral vector LMPd-ametrine, which permits sorting shRNA expressing cells based on ametrine expression by flow cytometry. These shRNAs were packaged into virus-like particles (VLPs), transduced into J-Lat 10.6 cells, and sorted for ametrine expression (Figure S11A). As expected, both *FUBP3* and HIV transcripts were significantly downregulated (Figures S11B and S11C). Similarly, the genes *CCR4*, *IL7R*, *RRM2*, *CDK1*, *KLHL8*, and *E2F2* were also significantly downregulated, as confirmed by RT-qPCR (Figure S11D). This validation strengthens our results by mitigating indirect effects of puromycin selection.

Interestingly, the transmembrane glycoprotein CD2, which is instrumental for driving T cell activation, was observed to be downregulated at both the mRNA and protein levels in all cell lines following FUBP3 knockdown (Figure S12). CD2 is a cell surface receptor expressed on T cells, NK cells, thymocytes, and dendritic cells and is commonly involved in T cell activating pathways through its interactions with lymphocyte function-associated antigen 3 (LFA3).^79^^,^^80^ Although CD2 has been identified as a hallmark of latently infected resting CD4^+^T cells in people living with HIV, its potential role in HIV-1 transcriptional activation has yet to be explored.^81^

Finally, given that we observed downregulated genes associated with T cell activation pathways, we pursued the identification of other pathways potentially regulated by FUBP3 that are implicated in inflammation. To achieve this, we used the fgsea package in R^82^ to conduct gene set enrichment analysis (GSEA). The normalized enrichment score (NES) reveals whether a particular gene set is enriched among the upregulated (light red) or downregulated (light blue) genes in each cellular model (Figure 3D). Interestingly, we discovered that the TNF-α signaling via NF-κB pathway was significantly downregulated following FUBP3 depletion in both J-Lat 10.6 cells and primary CD4^+^T cells, with a more modest downregulation observed in Jurkat cells (Figure 3D). Given that NF-κB is pivotal in initiating HIV-1 transcription, these findings suggest that FUBP3 may activate HIV-1 transcription through indirect mechanisms. Additionally, we observed significant downregulation of both interferon (IFN)-α and -γ responses in all three cellular models following FUBP3 depletion (Figure 3D). IFNs are crucial in activating the immune system’s protective defenses in response to viral infections.^83^^,^^84^ Intriguingly, this suggests that FUBP3 could also regulate the expression of these IFNs, potentially stimulating the immune response against HIV-1 replication *in vivo*.

In summary, FUBP3 regulates the expression of numerous genes involved in cancer regulation, as well as genes pertinent to T cell activation, cell cycle processes, and TNF-α-induced inflammation via the NF-κB pathway in J-Lat 10.6, uninfected Jurkat, and uninfected primary CD4^+^T cells. This suggests that FUBP3, in addition to potentially having a direct effect on HIV transcription, may also influence HIV-1 through its regulation of other cellular genes.

### FUBP3 is required for efficient HIV-1 transcription, acting independently of direct promoter binding

**Figure 4:**
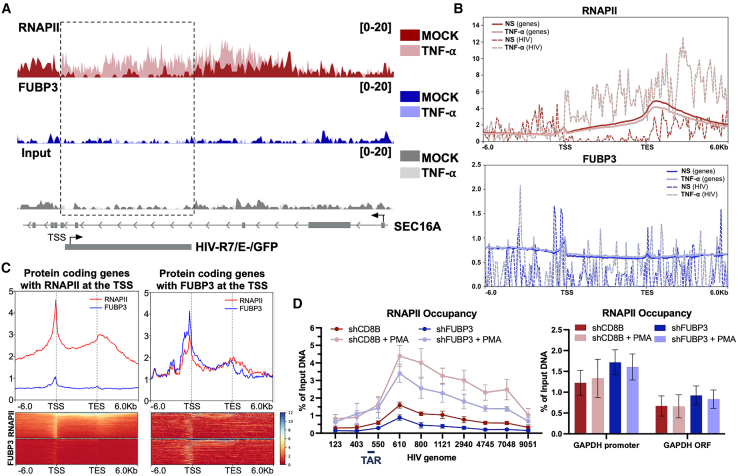
FUBP3 acts as a genomic transcription activator (A) Genome tracks of native ChIP-seq to RNAPII, FUBP3, and input in J-Lat 10.6 cells with or without stimulation (mock or TNF-⍺). (B) Scaled average normalized coverage of indicated factors at protein-coding genes >2 kb with expression levels comparable to HIV (1,790 genes) in J-Lat 10.6 cells in non-stimulated conditions (NS) or TNF-α stimulation. The dotted lines represent the coverage at the HIV-1 locus, and the solid lines represent the coverage of the cellular genes. (C) Scaled average normalized coverage of indicated factors at protein-coding genes with an RNAPII (left: 2,952 genes) or FUBP3 (right: 785 genes) peak at −2,000 to +300 of the TSS across indicated regions, binned in 6-kb windows. (D) RNAPII recruitment onto the HIV genome (left) or GAPDH (right) in Jurkat-D6 cells as determined by XChIP qPCR. Results are presented as percentage immunoprecipitated DNA over input, after IgG control background subtraction. All data are reported as the mean ± SEM.

To determine whether FUBP3 directly or indirectly binds to the HIV promoter and to investigate its relationship with RNAPII, we conducted native ChIP (NChIP) sequencing (NChIP-seq). Briefly, J-Lat 10.6 cells underwent either mock stimulation or treatment with TNF-α (10 ng/mL, 6 h) prior to nuclei isolation and MNase digestion (Figure S13A). Subsequently, RNAPII and FUBP3 were immunoprecipitated, and the associated genomic DNA was sequenced (Figure S13A). Under unstimulated conditions (under latency), residual RNAPII occupancy was observed on the HIV genome, as indicated by the dark red track in Figure 4A. As anticipated, upon TNF-α-induced reactivation of HIV, there was a significant increase in RNAPII occupancy across the HIV locus compared to the input (Figure 4A, light red track versus dark red and gray tracks). Although small peaks were detected upstream of the TSS, no significant FUBP3 occupancy was observed on the HIV genome in either mock-stimulated or TNF-α-treated conditions (Figures 4A and S13B, blue tracks) compared to the input (gray tracks). This suggests that FUBP3 may not directly interact with the HIV gene, but rather indirectly, possibly through a protein or RNA mediator. Additionally, native immunoprecipitation (IP) may not capture all interactions due to potential loss during the process. This contrasts with crosslinked ChIP (XChIP), which fixes weak interactions and prevents such loss, as was observed during ChAP-MS screening.

When examining cellular genes that are transcribed at levels equal to or greater than HIV (>60 TPM, *n* = 1790), a similar pattern of RNAPII occupancy was observed, characterized by a peak at the TSS followed by a pileup of RNAPII at the transcription end site (TES) (Figure 4B). A peak of FUBP3 occupancy was observed just upstream of the TSS, which then decreased back to baseline shortly after the TSS (Figure 4B). A similar pattern of occupancy for both RNAPII and FUBP3 was also noted across all protein-coding genes expressed in J-Lat 10.6 cells (Figure S13C).

A focused analysis of protein-coding genes with RNAPII bound either upstream of or at the TSS (*n* = 2,950) revealed a peak of FUBP3 occupancy just upstream of the TSS (Figure 4C). This suggests that a select number of genes transcribed by RNAPII are associated with FUBP3 (Figure 4C). Additionally, an analysis of genes with FUBP3 bound either upstream of or at the TSS (*n* = 785) showed that most genes occupied by FUBP3 are also occupied by RNAPII. Furthermore, this analysis demonstrated that FUBP3 strictly binds upstream of the TSS when it directly binds to DNA (Figure 4C).

To investigate FUBP3’s role in gene transcription activation, genomic regions were classified into four categories: active promoters, poised promoters, active enhancers, and poised enhancers. The analysis revealed that FUBP3 and RNAPII were primarily enriched at active promoters, with FUBP3 preferentially binding upstream of the TSS. Additionally, it showed minimal levels of occupancy at poised promoters (Figure S13D). This pattern supports the role of FUBP3 in transcriptional activation at specific genomic sites. Finally, the motif analysis of the genomic regions bound by FUBP3 revealed that FUBP3 shows binding affinity for AT-rich sequences that are similar to the FUSE^85^ (Figure S13E). Intriguingly, the top five sequences contained motifs like ATTC or TTTT, which were previously identified as having a high binding affinity with FUBP3 and other FBP proteins.^86^^,^^87^ However, these sequences could not be linked to specific genes, likely due to their location within non-coding regions. Collectively, these findings suggest that FUBP3 may act as a positive regulator of gene expression by binding in a FUSE-like manner upstream of the TSS. Subsequently, depletion studies of FUBP3 or CD8B control were carried out to evaluate the impact of FUBP3 on RNAPII occupancy at the HIV locus. XChIP qPCR assays were conducted on Jurkat-D6 cells following transduction with their respective shRNAs, with or without PMA treatment (10 nM, 6 h), and this was followed by IP of RNAPII. Although no change in RNAPII occupancy was observed before the TSS of the HIV promoter, a reduction in RNAPII recruitment and elongation was noted, beginning between Nuc-1 (∼550 nt) and Nuc-2 (∼800 nt), resulting in an overall decrease in RNAPII elongation across the HIV genome (Figure 4D). Notably, RNAPII recruitment on the promoter and open reading frame (ORF) of the housekeeping gene *GAPDH* was not affected (Figure 4D).

In summary, these results suggest that, while FUBP3 does not bind directly to the HIV-1 promoter, it is essential for HIV-1 transcription processing, as elongating RNAPII occupancy is reduced upon FUBP3 depletion.

### FUBP3 interacts with and stabilizes Tat to promote HIV-1 transcription

**Figure 5:**
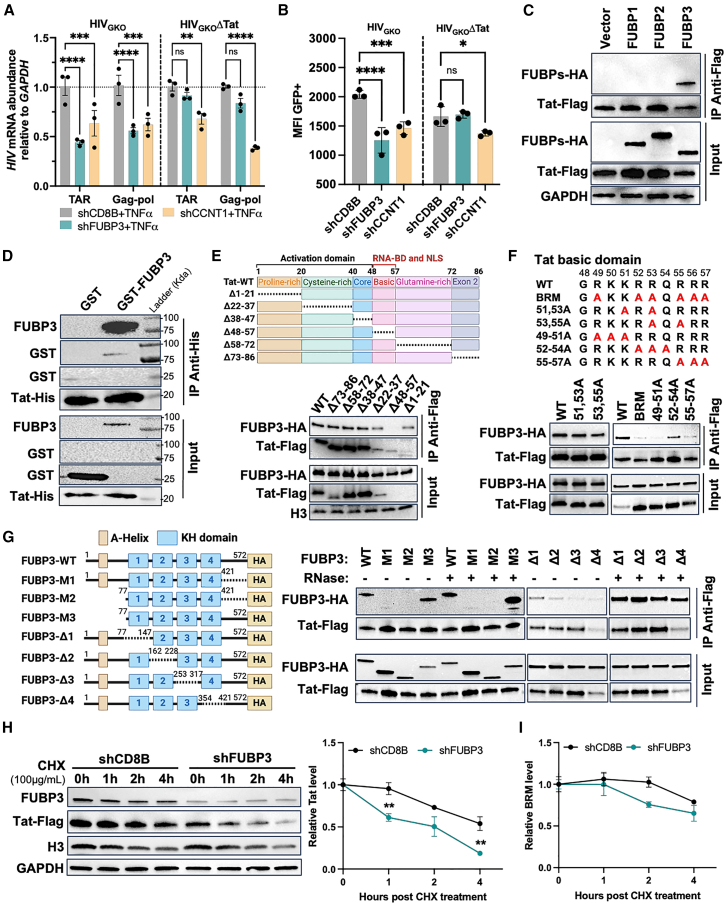
FUBP3 regulates HIV-1 in a Tat-dependent manner (A) *HIV* mRNA abundance upon FUBP3 or CCNT1 depletion in HIV_GKO_/HIV_GKO_ΔTat clone cells as determined by RT-qPCR. (B) Representative flow cytometry plot showing expression of HIV (MFI GFP+). (C) Tat-FUBPs coIP from HEK293T cells expressing Tat-FLAG and FUBPs-HA. (D) *In vitro* binding assay of GST-FUBP3 and His-Tat-WT protein. (E) Schematic representation of FLAG-Tat deletion variants and Tat-FUBP3 coIP from HEK293T cells expressing Tat-FLAG deletion variants and FUBP3-HA. (F) Schematic of the mutated Tat basic domain variants and Tat-FUBP3 coIP with Tat-FLAG mutated basic domain variants and FUBP3-HA. (G) Tat-FUBP3 coIP with Tat-FLAG and FUBP3-HA truncations, pretreated or not with RNase before IP. (H) Tat-FLAG degradation assay with FUBP3 or CD8B deletion quantified by western blot and RT-qPCR upon treatment with cycloheximide (CHX) in HEK293T cells. (I) Tat-BRM protein level quantification over time by western blot following CHX treatment. ∗*p* < 0.05, ∗∗*p* < 0.01, ∗∗∗*p* < 0.001, ∗∗∗∗*p* < 0.0001, as determined by two-way ANOVA with multiple comparisons. All data are reported as the mean ± SEM.

We then explored whether the enrichment of FUBP3, as observed on active promoters by ChAP-MS, is facilitated by the viral transactivator protein Tat. This was conducted using Jurkat cells infected with a Tat-deficient version of the HIV_GKO_ vector (HIV_GKO_ΔTat), made in house (Figure S14A). Briefly, HIV_GKO_ and HIV_GKO_ΔTat were cloned into pCMV-Tag2B cloning vector and used to generate viral particles pseudotyped with the pantropic VSV-G envelope to infect Jurkat cells. Infected cells were sorted by flow cytometry in two groups: mKO2^+^GFP^−^ (latently infected) and mKO2^+^GFP^+^ (actively infected). The sorted cells were cultured over 2–3 weeks, and surviving clones were isolated and characterized by flow cytometry, HIV integrated reverse PCR, and Sanger sequencing (Figure S14A). FUBP3, along with CCNT1 and CD8B, was depleted in both HIV_GKO_ and HIV_GKO_ΔTat infected Jurkat cell clones (Figure S14B). HIV expression was then assessed to evaluate the impact of FUBP3 depletion. As expected, knocking down FUBP3 statistically significantly reduced both basal and TNF-α-induced transcription in HIV_GKO_ infected cells as measured by RT-qPCR (Figures 5A and S14C). Interestingly, in Jurkat cells infected with HIV_GKO_ΔTat, the knockdown of FUBP3 had little to no effect, suggesting that FUBP3 serves as a transcriptional activator of HIV-1 in a Tat-dependent manner (Figures 5A and S14C). This observation was further supported by flow cytometry results, which demonstrated a statistically significant reduction in GFP expression exclusively in HIV_GKO_ Jurkat cells following FUBP3 depletion (Figures 5B and S14D).

Based on these findings, we next examined whether FUBP3 interacts directly with Tat. IP using an anti-FLAG antibody was performed with cell lysates from HEK293T cells expressing Tat-FLAG and FUBP1-HA, FUBP2-HA, or FUBP3-HA tagged proteins. Western blot analysis revealed that only FUBP3 was enriched in the IP, indicating a specific interaction with Tat, while no significant binding was observed for FUBP1 or FUBP2 (Figure 5C). To determine if this interaction is direct, we expressed and purified recombinant His-tagged Tat and GST-tagged FUBP3 proteins. These proteins were incubated together followed by IP using an anti-His antibody. Western blot analysis confirmed that FUBP3 was strongly enriched in the IP, providing clear evidence of a direct interaction (Figure 5D). Although the signal in the input sample was relatively weak with the anti-GST antibody due to instrument sensitivity and antibody avidity, a distinct band was observed in the IP sample, where the FUBP3 protein is more concentrated (Figure 5D). To further investigate the interaction between Tat and FUBP3, we generated a series of Tat deletion variants and carried out coIP studies with overexpressed FUBP3-HA (Figure 5E). We observed reduced protein expression with the TatΔ1-21 aa and TatΔ48-57 aa variants, which may be attributed to issues with protein stability (Figure 5E). Although Tat’s interaction with FUBP3 remained stable across various Tat variants, including TatΔ1–21, a substantial reduction in interaction was observed with the TatΔ48–57 aa variant. This suggests that FUBP3 primarily binds to the basic domain of Tat (Figure 5E). Further experiments involving mutations within the basic domain of Tat (Figure 5F), highlighted specific amino acids, 49–51 and 55–57 aa, as essential for the interaction with FUBP3. Mutating these residues to alanine significantly diminished the Tat/FUBP3 interactions (Figure 5F).

Next, we constructed deletion variants of FUBP3 to pinpoint the region responsible for interacting to Tat (Figure 5G). The FUBP3 variant M3, which spans amino acids 77–572, showed enrichment similar to that of wild-type FUBP3-WT when coIP with Tat. This indicates that FUBP3 does not need the A-Helix domain to interact with Tat (Figure 5G). Deletion of amino acids 421–572 observed in the FUBP3-M1 and FUBP3-M2 variants, however, led to a significant loss of interaction, showing the importance of this specific region for the interaction with Tat (Figure 5G). All K-homology (KH) domain deletion variants of FUBP3 (FUBP3-Δ1–Δ4) displayed modest enrichment, suggesting that interactions with Tat are maintained across all domains to some extent. Interestingly, RNase treatment prior to IP led to higher enrichment for all variants that originally interacted with Tat (Figure 5G). This may be a consequence of elevated FUBP3 availability. As an RNA-binding protein, it is believed that FUBP3 interacts with a range of RNAs within the cell and treating with RNase likely liberates it from these RNA interactions, thus more FUBP3 is available for binding to Tat.

To delve deeper into the FUBP3-Tat interaction, we conducted a Tat stabilization assay as previously done.^35^ HEK293T cells were transfected with either Tat-FLAG or the Tat-BRM-FLAG mutant and subsequently treated with shRNAs targeting FUBP3 or CD8B as controls (Figures S15A and S15B). After transfection, the cells were treated with cycloheximide (CHX) to inhibit protein synthesis, allowing us to monitor the degradation of Tat protein over a 4-h period. Notably, protein levels at t = 0 h were used as a reference point to facilitate comparison of Tat stability in cells depleted of FUBP3 or CD8B. While FUBP3-deficient cells showed marked reduction of Tat protein half-life as compared to shCD8B control, no significant changes were seen at the mRNA level (Figures 5H and S15C). Moreover, we found that depletion of FUBP3 had no statistical effect on the stability of Tat-BRM or histone H3 as compared to the CD8B control (Figures 5I, S15D, and S15E). Collectively, these findings indicate that FUBP3 can specifically interact with the basic domain of Tat, and thereby increases its stability, likely leading to the enhancement of Tat-mediated transcription activation.

### FUBP3 directly interacts with and stabilizes TAR-RNA

**Figure 6:**
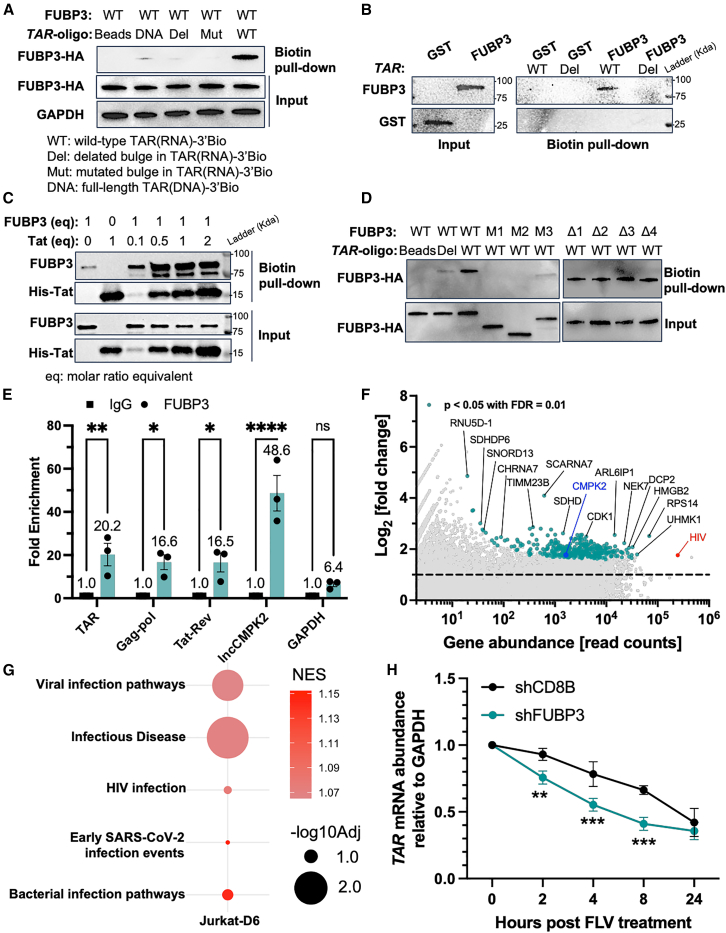
FUBP3 interacts with and stabilizes *TAR*-RNA (A) Immunoblotting of the *in vitro TAR*-RNA pull-down with FUBP3. (B) Immunoblotting of the *in vitro TAR*-RNA pull-down with GST-FUBP3. (C) Immunoblotting of the competitive *in vitro TAR*-RNA pull-down with GST-FUBP3 and His-Tat protein. (D) Immunoblotting of the *in vitro TAR*-RNA pull-down with FUBP3 variants. (E) RIP against control IgG or FUBP3 were subjected to qPCR to measure HIV mRNA level in Jurkat-D6 cells. (F) RIP samples were subjected to RNA-seq to reveal mRNAs bound by FUBP3 transcriptome-wide. (G) Bubble plot of GSEA results from the RIP-seq data. NES: normalized enrichment score. (H) *TAR*-RNA degradation with FUBP3 or CD8B deletion monitored by RT-qPCR over time upon treatment with flavopiridol in J-Lat 10.6 cells. ∗*p* < 0.05, ∗∗*p* < 0.01, ∗∗∗*p* < 0.001, ∗∗∗∗*p* < 0.0001, as determined by two-way ANOVA with multiple comparisons. All data are reported as the mean ± SEM.

As illustrated, FUBP3 interacts with the basic domain of Tat, where binding to *TAR*-RNA is essential. Since FUBP3 binds to RNA, we investigated whether FUBP3 directly binds *TAR*-RNA and forms a complex with Tat. To explore this, we performed a pull-down assay as previously described^88^ using biotin-labeled *TAR*-RNA (*TAR*-WT) and lysates from HEK293T cells transfected with FUBP3-HA (Figure S16A). A significant enrichment of FUBP3 was observed following the pull-down with *TAR*-WT. In contracts, only minimal interaction was observed either when the bulge of *TAR*-RNA was removed (*TAR*-Del), mutated to adenine nucleotides (*TAR*-Mut), or when *TAR* was in DNA (*TAR*-DNA) form (Figure 6A). This suggests that the structure, sequence, and form of *TAR* are crucial for its interaction with FUBP3. As a control, we conducted a similar experiment using HEK293T cells transfected with Tat-FLAG. The outcomes were consistent with those for FUBP3 and as described in the literature,^88^ exhibiting a strong enrichment of Tat with *TAR*-WT and very weak to no interactions with *TAR*-Del, *TAR*-Mut, and *TAR*-DNA (Figure S16B). As expected, the Tat mutant with six-point mutations in the basic domain (Tat-BRM) did not interact with *TAR* (Figure S16B). This also highlights the importance of the basic domain in mediating interactions with *TAR*. We next wanted to see if FUBP3 and *TAR* interact directly or through an intermediary protein. To do this, we incubated purified recombinant FUBP3-GST *in vitro* with either *TAR*-WT or *TAR*-Del, followed by a biotin/streptavidin pull-down assay. Using *TAR*-WT, we detected enrichment of FUBP3, but not with *TAR*-Del, confirming that the interaction between FUBP3 and *TAR* is direct and specific (Figure 6B).

Considering that FUBP3 and Tat appear to interact mutually with *TAR* and each other via similar regions, we speculated that these interactions may be competitive. To test this possible competitive dynamic, we performed co-transfections of FUBP3-HA and Tat-FLAG together with either positive binders to *TAR* (Tat-WT and FUBP3-WT) or negative binders (Tat-BRM and FUBP3-M1), followed by a biotin pull-down assay. Interestingly, the pull-down efficiency was not significantly different across both positive and negative binder combinations for FUBP3 and Tat, indicating no significant competitive effect between FUBP3 and Tat in their binding to *TAR* (Figure S16C). The only apparent difference was a decrease in the co-pull-down of cofactors CCNT1 and CDK9 using Tat-BRM. As a mutant that fails to bind *TAR*, Tat-BRM also reduces overall binding efficiency of the complex with these cofactors (Figure S16C). Overall, these results suggest that FUBP3 and Tat can bind *TAR* independently of one another and do not significantly hinder one another’s binding, displaying non-competitive or possibly cooperative interactions at this regulatory element. To confirm the interactions of FUBP3, *TAR*, and Tat, we performed an *in vitro* binding competition assay. This was done with recombinant GST-FUBP3 and His-Tat protein and biotinylated *TAR*-RNA. Consistent with previous observations (Figure S16C) competitive inhibition of *TAR* binding did not occur between FUBP3 and Tat (Figure 6C). Remarkably, though, Tat dramatically increased the binding affinity of FUBP3 to *TAR* in a dose-dependent manner (Figure 6C). This finding suggests a synergistic mechanism where Tat potentially recruits FUBP3 to *TAR*, enhancing RNA stability and thereby promoting transcriptional activity.

To pinpoint the exact domain of FUBP3 that associates with *TAR*-RNA, we employed previously generated variants of FUBP3 (Figure 5G) in pull-down assays using biotinylated *TAR* (Figure 6D). Deletion of the sequence encompassing amino acids 421–572 completely abolished the interaction between FUBP3 and *TAR* (FUBP3-M1 and -M2), whereas deletion of the sequence encompassing the first 1–77 amino acids led to a partial loss of the interaction between these two proteins (FUBP3-M2 and -M3). Such results highlight the important roles these regions play in *TAR*-RNA binding. In addition, deletion of each KH-domain of FUBP3 did not alter binding to *TAR*-RNA, indicating no individual contribution of each KH-domain to *TAR*-RNA. Rather, they could all contribute to the interaction, meaning that the loss of a single domain is not enough to disrupt the binding (Figure 6D). Indeed, future experiments with specific point mutations in these domains could further clarify this interaction, helping to delineate more precisely the contributions of each domain to *TAR*-RNA binding.

To further probe whether FUBP3 can bind to *TAR*-RNA and other HIV mRNAs in a cellular context, we conducted RNA-binding protein IP (RIP) experiments followed by RT-qPCR in Jurkat-D6 cells that had been stimulated with TNF-α (10 ng/mL) overnight (Figure S17A). This assay showed a significant association with several HIV mRNAs, such as *TAR*, *Gag-pol*, and *Tat-Rev*, containing the *TAR*-RNA sequence. Additionally, FUBP3 was also found to interact with the previously reported long non-coding RNA lnc-*CMPK2*.^45^ Importantly, no interaction was observed with the housekeeping gene *GAPDH* (Figure 6E). This pattern suggests a selective binding of FUBP3 to specific RNA sequences or secondary structures, notably those involved in HIV transcription and regulation. To explore FUBP3/RNA interactions in Jurkat-D6 cells, RIP samples were sequenced. As an RNA-binding protein, there was an expectation that FUBP3 would bind a wide range of RNAs. As anticipated, the sequencing data confirmed this expectation, revealing that FUBP3 associated with a total of 346 RNAs, demonstrating an extensive RNA-binding profile (Figure 6F). Also as expected, the sequencing results showed a clear enrichment of HIV mRNA along with the positive control *CMPK2*, while *GAPDH* was not detected in the samples (Figure 6F). Interestingly, one of the RNAs bound by FUBP3 implicated in HIV-1 infection processes is CDK1, which also shows significant downregulation upon FUBP3 depletion (Figures 6F and S10). This finding was substantiated through qPCR validations (Figure S17B). CDK1 is known to promote HIV-1 reverse transcription in activated CD4^+^T cells and various transformed cell lines. It is hypothesized to impact HIV-1 replication by phosphorylating SAMHD1, a recognized inhibitor of HIV-1 replication.^78^^,^^89^^,^^90^^,^^91^ Given this context, FUBP3’s interaction with *CDK1* mRNA may suggest a broader role where FUBP3 facilitates modulation of protein expression that indirectly influences HIV replication dynamics. To better understand the biological implications of these interactions, we conducted GSEA using the fgsea package in R.^82^ This revealed marked enrichment of RNAs that translate to proteins that function in inflammatory pathways, most notably TNF-α signaling through NF-κB, and IFN-α signaling, consistent with our previous RNA sequencing (RNA-seq) study (Figure S17C). Additionally, a number of FUBP3-bound RNAs target were implicated with cancer-related pathways, including E2F targets, mTORC1 signaling, carcinoma, and leukemia; as well as cell cycle regulation processes such as mitotic progression and the G2/M checkpoint (Figure S17C). Interestingly, a pathway related to Alzheimer’s disease was also enriched, aligning with a recent study suggesting a potential link between FUBP3 and neurodegeneration (Figure S17C).^92^ Most importantly, FUBP3 was found to interact with RNAs encoding proteins involved in viral infections, including HIV, as well as other infectious diseases (Figure 6G). These findings suggest that FUBP3 may have a wide-ranging regulatory influence on cellular processes via its RNA-binding activity. Future research should investigate how FUBP3 interacts with these RNAs and whether it directly modulates these pathways or functions indirectly, particularly in the context of HIV pathogenesis.

To investigate the significance of the FUBP3/*TAR*-RNA interaction, we used J-Lat 10.6 cells where either FUBP3 or CD8B control was depleted using shRNAs (Figure S18A). The cells were subsequently treated with flavopiridol (FLV), a transcription inhibitor, allowing us to track the degradation rate of pre-existing *TAR*-RNA over time. Without FUBP3, *TAR*-RNA degraded at an accelerated rate compared to shCD8B control cells. This implies that FUBP3 plays a crucial role in stabilizing *TAR*-RNA (Figure 6 h). Cell viability remained unaffected during flavopiridol treatment (Figure S18B). Importantly, the same experiment carried out in J-Lat 10.6 cells lacking the viral Tat protein^93^ showed no significant effect on *TAR*-RNA stability upon FUBP3 knockdown, emphasizing the Tat-dependent mechanism by which FUBP3 stabilizes *TAR*-RNA (Figures S18C and S18D). To determine whether RNA stabilization is a general function of FUBP3, we applied the same protocol to other genes. Specifically, we examined the stability of *RRM2*, *CDK1*, and *KLHL8* mRNAs, which are significantly downregulated upon FUBP3 depletion in J-Lat 10.6 cells and are bound by FUBP3 at the mRNA level in Jurkat-D6 cells. The results showed that *RRM2* mRNA degraded more rapidly in the absence of FUBP3. However, the degradation of *CDK1* and *KLHL8* were similar regardless of the presence or the absence of FUBP3 (Figure S19A). Additionally, we assessed the stability of *CMPK2* mRNA, which is bound by FUBP3 but not regulated by it; *E2F2* mRNA, which is not bound by FUBP3 but significantly downregulated upon its depletion; and *PAK1* mRNA, which is neither bound nor regulated by FUBP3. In all cases, these mRNAs did not exhibit differential degradation regardless of FUBP3 depletion (Figures S19B–S19D). Together, these observations imply that, while FUBP3 has the capacity to stabilize certain RNAs, its role in RNA stabilization is not a general biological mechanism across all transcripts it interacts with.

## Discussion

In this study, we have elucidated the mechanism by which FUBP3 regulates HIV-1 transcription, revealing interactions with the viral transactivator Tat and with its cognate *TAR*-RNA. Our results show that FUBP3 is a positive regulator of HIV-1 transcription, as FUBP3 depletion leads to decreased expression of HIV in a Tat-dependent manner (Figures 1, 2, 5A, and 5B). By using various biochemical assays and transcriptomic analyses, we provide compelling evidence that FUBP3 plays a crucial role in maintaining HIV-1 transcriptional activity (Figures 3 and 4).

The interaction between FUBP3 and the basic domain of Tat underscores a key mechanism in HIV-1 regulation, as this domain mediates the binding of Tat to *TAR*-RNA. Our data suggest that FUBP3 enhances the stability of Tat (Figure 5), extending its half-life, critical for recruiting the transcriptional machinery, P-TEFb, to the HIV promoter.^94^^,^^95^ The specific enrichment of FUBP3 with *TAR*-RNA also supports the model that FUBP3 functions as a cofactor to promote transcription from the HIV-1 promoter.

**Figure 7:**
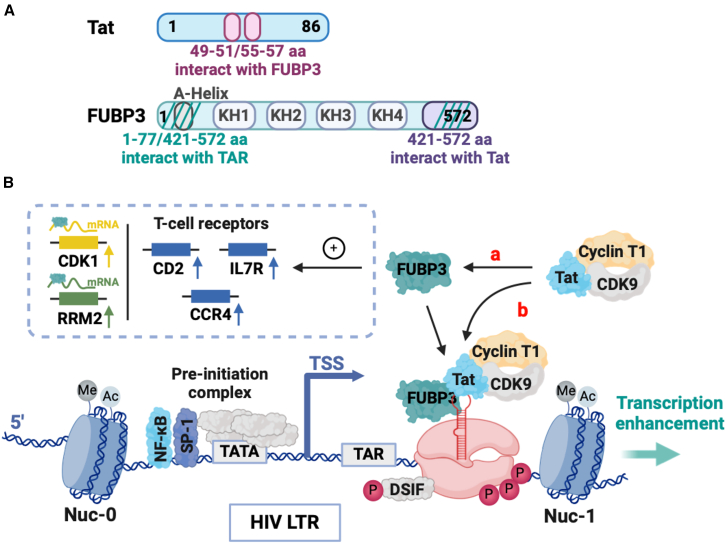
Model of FUBP3’s role in HIV transcriptional regulation (A) Summary of Tat and FUBP3 interactions with each other and FUBP3 with *TAR*. (B) FUBP3 binds and stabilizes *TAR* and Tat, promoting HIV transcription. (a) FUBP3 binds and stabilizes Tat. FUBP3 bound to Tat binds and stabilizes *TAR*. (b) Tat’s complex binds to *TAR* and FUBP3, already stabilizing the RNA. FUBP3 also regulates the expression of several T cell receptors as well as CDK1 and RRM2. FUBP3 binds CDK1 and RRM2 mRNAs and stabilizes RRM2 mRNA.

Importantly, we characterized FUBP3 binding preferences and found a strong affinity for *TAR*-RNA secondary structure that was diminished by deletion or mutations on this viral RNA (Figure 6). Importantly, this specificity implies that FUBP3 binds and stabilizes a specific conformation of *TAR*-RNA, which promotes the activity of Tat. Furthermore, Tat amplifies FUBP3’s binding to *TAR*-RNA, suggesting that Tat facilitates FUBP3 recruitment to stabilize the transcriptional complex (Figure 6C). The ability of FUBP3 to bind and stabilize *TAR*-RNA and Tat appear to be the mechanism by which it regulates HIV-1 transcription (Figure 7). Without this stabilization, the Tat-*TAR* interaction may be weakened, reducing the efficiency of P-TEFb recruitment and consequently impairing RNAPII elongation. Our data suggest that the formation of a stable Tat-*TAR*-FUBP3 complex amplifies transcriptional activation at the HIV promoter. However, the precise chronological order of binding between these components remains to be investigated.

Our study also showed that FUBP3 binds to a subset of active promoters upstream of the TSS, suggesting a wider role in modulating cellular transcriptional states (Figures 4C and S13D). This is consistent with our results that FUBP3 may also regulate gene expression independently of its role on HIV transcription, including modulation of pathways associated with T cell activation and inflammatory responses. Indeed, FUBP3 depletion led to significant dysregulation of many genes involved in immune response (e.g., *TNFAIP3*, *IER3*, *CD83*), TFs (e.g., *E2F2*, *ELF4*, *PROX1*), T cell receptors (e.g., *CD2*, *IL7R*, *PTPRC*), and oncogenic pathways (e.g., *RRM2*, *CDK1*, *CDKN1A*), demonstrating its broader regulatory function (Figures 3B and 3C). Genes implicated in TNF signaling pathway and IFN α and IFN γ responses were downregulated, indicating FUBP3’s role in modulating inflammatory responses. For instance, more recently FUBP3 has been implicated in neuron-mediated immune responses in Alzheimer’s disease^92^ and shown to positively regulate type-I IFN (IFN-I) signaling,^96^ further underscoring its importance in immune regulation.

The interaction between FUBP3 and HIV-1 mRNA, as demonstrated by RIP assays (Figures 6E and 6F), highlights its role in post-transcriptional regulation. Furthermore, we show that FUBP3 directly binds and stabilized the HIV *TAR*-RNA, consistent with a role in RNA stability (Figure 6 h). The identification of 346 RNAs associated with FUBP3 suggests that it may also influence cellular responses on a broader scale, including in viral infections where FUBP3 could impact the stability and replication of viral mRNA as well as the host cell response (Figure 6G).

Although our findings reveal an important role for FUBP3 in HIV-1 transcription, several questions remain. Future studies should evaluate its broader impact on RNA stability and explore its function throughout the viral life cycle. The specific mechanisms by which FUBP3 modulates immune response and T cell activation require further investigation. While our results do not indicate that FUBP3 influences the efficiency of HIV integration, we did not rule out changes in integration site preferences (e.g., euchromatin versus heterochromatin). Finally, exploring the therapeutic potential of targeting the HIV-1/FUBP3-mediated pathways could lead to innovative strategies for HIV-1 treatment.

Overall, our study identifies FUBP3 as a key player in the transcriptional regulation of HIV-1, enhancing our understanding of the intricate interplay between viral and host factors at the molecular level. By revealing the diverse roles of FUPB3, our findings lay a solid foundation for future research aimed at exploring its potential as a target for HIV-1 therapies. Furthermore, our findings highlight FUBP3’s possible involvement in oncogenic or inflammatory pathways, underscoring its significance beyond viral infections. This provides groundwork for future studies that could lead to innovative therapeutic strategies not only to fight HIV but also for other related pathologies.

## Materials and methods

### Cell lines and cell culture conditions

Jurkat, Jurkat-D6, J-Lat 10.6, and J-Lat 10.6 Tat-KO (a gift from Dr. Iván D’Orso) cells were cultured in RPMI (Gibco, #11875-119) supplemented with [v/v] 5% FBS (Atlas Biologicals #FS-0500-AD) and [v/v] 1% penicillin/streptomycin (Gibco #10378-016). Viral reactivation was achieved with cells treated with TNF-α (10 ng/mL, Sigma-Aldrich #T6674), TSA (Sigma-Aldrich #T-1952), SAHA (2.5 μM, LC Laboratories #V-8477), or PMA (10 nM, Fisher #BP6851). Cell confluency and viability were monitored by trypan blue staining.

### Plasmids

FUBP3-HA-WT was initially constructed in a pcDNA3.1 vector and was then used as a template for deletions (Table S1). FUBP3 constructs were then inserted in a pMSCV-IRES-mcherry vector (Addgene, #52114) and packaged with pCVM-VSV-G (Addgene #8454) and CMV intron (a gift from Greg Towers, UCL) for overexpression experiments. pMKO.1-puro (Addgene, #8452) was used for shRNA (targeting *CCNT1*, *FUBP1*, *FUBP2*, *FUBP3*, and *CD8B*, Table S1) cloning and was packaged in HEK293T cells for pseudotyped retrovirus with pUMVC3 (Addgene #8449) and pCVM-VSV-G (Addgene #8454). LMPd-ametrine vector shRNAmirs (targeting *FUBP3* and *CD19*, Table S1) were packaged for pseudotyped retrovirus in HEK293T cells with pCVM-VSV-G (Addgene #8454) and CMV-intron (a gift from Dr. François-Loïc Cosset). pFI-Tat(86)-FLAG-WT, pFI-Tat(86)-FLAG-BRM, and other Tat mutation/deletions constructs were generated as previously described.^35^^,^^53^

### Primary cell culture and transduction

PBMCs were first separated from blood from three healthy donors (LifeSouth Community Blood Center) using Ficoll-Paque Plus (Cytiva, #17144002). Then, CD4^+^T cells were isolated from PBMCs by negative selection (EasySep Human CD4^+^T Cell Enrichment kit, Stemcell Technologies, #19052) and activated with Dynabeads Human T activator CD3/CD8 (Gibco, #11131D) in T cell medium (RPMI, Gibco #11875-119; [v/v] 1% MEM vitamin solution, Gibco #11120-052; [v/v] 1% non-essential amino acids, Gibco #11140-050; [v/v] 1% sodium pyruvate, Gibco #11360-070; [v/v] 1% L-arginine/L-asparagine/folic acid, Sigma-Aldrich #A5006/#A4159/#F8758; [v/v] 1% HEPES pH8, Gibco #15630-080; [v/v] 1% penicillin/streptomycin, Gibco #10378-016; [v/v] 5% FBS, Atlas Biologicals #FS-0500-AD; 6 ng/mL human interleukin-2, Miltenyi Biotec #130-097-748; 36 ng/mL human interleukin-7, PeproTech #200-07; 36 ng/mL human interleukin-15, PeproTech #200-15). Three days after activation, donors 1, 2, and 3 were transduced with pMKO-puro vector packaged with pUMVC3 (Addgene #8449) and pCVM-VSV-G (Addgene #8454) expressing shCD8, shFUBP3, or shCCNT1, followed by selection with puromycin (1.5 μg/mL) for 3 days. Cells were then infected overnight with NL4-3 at 5 ng/million cells. All donors were washed 16 h following infection and maintained in culture for 16 days. Cell viability and cell number was monitored by trypan blue staining. On day 13, cells were pelleted for RNA quantification western blot analysis, and medium was collected for p24 viral protein expression.

### RT-qPCR analysis

Total RNA was harvested using RNeasy kit (Qiagen, #74106) followed by DNase treatment using TURBO DNase (Invitrogen, #AM2239) per the manufacturer’s recommended protocol. Approximately 500 ng of total RNA was reverse transcribed using random primers with the SuperScript III kit (Invitrogen, #18080-051) in 20 μL of total reaction volume per the vendor’s recommended protocol. Then, 2 μL of cDNA was used for qPCR for each pair of primers (Table S2) with SensiFAST SYBR No-ROX kit (Meridian, #BIO-98020). The qPCR reaction was then aliquoted into three technical replicates (10 μL) per biological replicate and analyzed by a Bio-Rad CFX384 real-time system. Relative expression level of each transcript was determined by normalizing to the housekeeping gene (*GAPDH* or *RPL13A*) unless indicated otherwise using the 2^−ΔΔCt^ method.^97^

### HIV DNA integration assay

Genomic DNA was extracted from 500,000 cells the day following infection with the NL4-3 HIV virus using the DNeasy blood Tissue kit (Qiagen, #69506) per the manufacturer’s recommended protocol. One first PCR was then run with 50 ng of gDNA using 0.3 μL of DreamTaq Hot Start DNA polymerase (Thermo Fisher, #EP1701), 5 μL of 10× DreamTaq buffer, dNTP (100 μM), and the corresponding Alu primers (Table S2; F, 1.2 μM and R, 0.2 μM) in 50-μL PCR final volume. The following cycling conditions were used: 94°C for 2 min, 20 cycles of 94°C for 20 s, 50°C for 10 s, and 65°C for 3.5 min, followed by a final extension at 65°C for 7 min. The provirus integration was then quantified by qPCR with primers in the LTR (MSS: 0.25 μM), 2 μL of 1/30 dilution of Alu-PCR amplification and SensiFAST SYBR No-ROX kit (Meridian, #BIO-98020). Relative integration was determined by normalizing to the housekeeping gene (*GAPDH*) using the 2^−ΔΔCt^ method.^97^

### p24 quantification by ELISA

Upon infection with NL4-3, medium was collected at different time point and cells were counted by trypan blue staining as described above. Quantification of p24 was performed by ELISA using the HIV-1 p24 Antigen Capture Assay (ABL, #5447) per the manufacturer’s recommended protocol. Viral protein quantification was finally normalized to the cell number at the time of medium collection.

### Western blot

Cells were lysed with RIPA buffer (10 mM Tris-HCl pH 8, 1 mM EDTA, 140 mM NaCl, [v/v] 1% Triton, [v/v] 0.1% SDS, [m/v] 0.1% Na deoxycholate) supplemented with complete ULTRA protease inhibitor cocktail tablets (Roche, #05892791001) on ice for 15 min. Then, the lysate was centrifuged at 13,000 × *g* for 10 min at 4°C. Protein concentration in the supernatant was evaluated by Bradford assay (Bio-Rad, #5000006). Total protein extracts were resolved on a precast 4%–20% SDS-PAGE gel (Bio-Rad, #4568094) in 1× Tris-glycine-SDS buffer (AG Scientific, #T-2711) and transferred onto a nitrocellulose membrane (Bio-Rad, #1704270) using a Bio-Rad Trans-Blot Turbo Transfer System. Membranes were probed with GAPDH (Santa Cruz, #sc-32233), Histone3 (EMD Millipore, #07–690), FUBP3 (Abcam, #ab181025), CCNT1 (Thermo Fisher, #PA577892), CD2 (Cell Signaling, #53460S), RRM2 (Abcam, #ab57653), RORC (Thermo Fisher, #14-6988-82), RNAPII (EMD Millipore, #05–623), CDK9 (Proteintech,#11705-1-AP), DSIF (Proteintech, #16511-1-AP), anti-HA (Millipore Sigma, #H9658), and anti-FLAG (Millipore Sigma, #F3165) followed by anti-mouse IgG (Cytiva, #NA931V) or anti-rabbit IgG (Cytiva, #NA9340V). The membranes were exposed with ECL Prime Western Blotting Detection Reagent (Cytiva, #RPN2236), imaged using the Bio-Rad Gel Doc XRS+ Molecular Imager and analyzed by Image Lab software (Bio-Rad).

### RNA-seq

Total RNA was harvested and DNase treated as described above. NEB Ultra II Kit with ribosomal RNA depletion was used to prepare the RNA-seq libraries, and libraries were sequenced in the NextSeq 500 v2 using paired end, 2 × 40 kits. The raw .fastq files were aligned to a custom human genome (Hg38) supplemented with the HIV gene using STAR.^98^ Gene expression changes were estimated with Featurecounts^99^ and Deseq2.^100^ Protein interactions pathways were analyzed using STRING db v12.^101^ The fgsea package was used to conduct gene pathway analysis.^82^^,^^102^^,^^103^^,^^104^

### X-ChIP assay

As previously described,^35^ cells were crosslinked for 10 min with 1% formaldehyde and quenched with 0.125 M glycine for 5 min at room temperature. Pellets of 10 million cells were resuspended in SDS-lysis buffer (50 mM Tris-HCl pH 8, 1% [v/v] SDS, 10 mM EDTA, supplemented with proteinase inhibitor) and sonicated 18 times for 10-s bursts on ice to generate sheared chromatin of length 200–400 bp. A total of 500 μg of protein was used for each IP with antibody FUBP3 (Abcam, #ab181025), RNAPII (EMD Millipore, #05-623), and mouse IgG (Invitrogen, #31903) in ChIP dilution buffer (16.7 mM Tris-HCl pH 8, 1.2 mM EDTA, 0.01% SDS, 1.1% Triton X-100, 167 mM NaCl). The equivalent of 1% chromatin was saved as input control. DNA was immunoprecipitated against Dynabeads Protein G (Invitrogen, #10004D) and washed first with 1 mL of low-salt wash buffer (20 mM Tris-HCl pH 8, 150 mM NaCl, 2 mM EDTA, [v/v] 1% Triton X-100, [v/v] 0.1% SDS), then 1 mL of high-salt wash buffer (20 mM Tris-HCl pH 8, 500 mM NaCl, 2 mM EDTA, [v/v] 1% Triton X-100, [v/v] 0.1% SDS), 1 mL of LiCl wash buffer (10 mM Tris-HCl pH 8, 1 mM EDTA, 250 mM LiCl, [v/v] 1% NP-40, 1 mM deoxycholic acid), and finally 1 mL of TE buffer (10 mM Tris-HCl pH 8, 1 mM EDTA). Samples were treated with RNase A (Thermo Scientific, #EN0531) for 30 min at 37°C, reversed crosslinked with 200 mM NaCl for 4h at 65°C, and treated with proteinase K (Thermo Scientific, #EO0491) for 1 h at 60°C. The DNA was purified using the QIAquick PCR purification kit (Qiagen, #28106) and analyzed by qPCR using the primers listed in Table S3. The IgG background was subtracted, and the datasets were normalized to input values as follows:

$$percent\;input=2^{{CT}_{input}-{CT}_{IP}}\times 100$$

### N-ChIP assay

Cells were resuspended in 1 mL of buffer 1 (15 mM Tris-HCl pH 8, 60 mM KCl, 15 mM NaCl, 5 mM MgCl_2_, 0.1 mM EDTA, 0.3 M sucrose, 5 mM sodium butyrate, 0.1 mM PMSF, 0.5 mM DTT) and lysed with addition of 1 mL of buffer 2 (buffer 1 supplemented with [v/v] 0.8% NP-40) for 7 min on ice. Then, 8 mL of buffer 3 (15 mM Tris-HCl pH 8, 60 mM KCl, 15 mM NaCl, 5 mM MgCl_2_, 0.1 mM EDTA, 1.2 M sucrose, 5 mM sodium butyrate, 0.1 mM PMSF, 0.5 mM DTT) were added and nuclei were pelleted at 2,000 × *g* for 20 min at 4°C. Supernatant was removed and nuclei were resuspended in 0.5 mL of MNase digestion buffer (50 mM Tris-HCl pH 8, 0.3 M sucrose, 0.2 mM PMSF, 4 mM MgCl_2_, 1 mM CaCl_2_, 5 mM sodium butyrate). MNase enzyme (Biolabs, #M0247S) was added and DNA was digested for 5 min at 37°C. The digestion was quenched with 20 μL of 0.5 M EDTA and samples were centrifuged at 14,800 × *g* for 10 min at 4°C. The supernatant S1 was kept at −80°C and the pellet was dialyzed using slide-a-lyzer (Thermo Fisher, #69550) in dialysis buffer (1 mM Tris-HCl pH 8, 0.2 mM PMSF, 5 mM sodium butyrate, 0.2 mM EDTA). The dialyzed product was centrifuged at 14,800 × *g* for 10 min at 4°C and the supernatant S2 was combined with S1. Samples were centrifuged two more times at 14,800 × *g* for 10 min at 4°C to remove cell debris and chromatin were quantified by nanodrop (260/280). A total of 30 μg of DNA was used for each IP with antibody FUBP3 (Abcam, #ab181025), RNAPII (EMD Millipore, #05-623) and mouse-IgG (Invitrogen, #31903) in antibody incubation buffer (20 mM Tris-HCl pH 8, 0.2 mM PMSF, 5 mM EDTA, 20 mM sodium butyrate, 150 mM NaCl). The equivalent of 4% chromatin was saved as input control. DNA was immunoprecipitated against Dynabeads Protein G (Invitrogen, #10004D) and washed first with 1 mL of low-salt wash buffer (20 mM Tris-HCl pH 8, 150 mM NaCl, 2 mM EDTA, [v/v] 1% Triton X-100, [v/v] 0.1% SDS), then 1 mL of high-salt wash buffer (20 mM Tris-HCl pH 8, 500 mM NaCl, 2 mM EDTA, [v/v] 1% Triton X-100, [v/v] 0.1% SDS), 1 mL of LiCl wash buffer (10 mM Tris-HCl pH 8, 1 mM EDTA, 250 mM LiCl, [v/v] 1% NP-40, 1 mM deoxycholic acid), and finally 1 mL of TE buffer (10 mM Tris-HCl pH 8, 1 mM EDTA). Immunoprecipitated DNA was eluted in elution buffer (20 mM Tris-HCl pH 8, [v/v] 0.5% SDS, 50 mM NaCl, 20 mM sodium butyrate, 0.1 mM PMSF) at 37°C for 15 min. DNA samples were treated with RNase A (Thermo Scientific, #EN0531) for 30 min at 37°C and digested with proteinase K (Thermo Scientific, #EO0491) for 15 min at 56°C. The DNA was purified using the QIAquick PCR purification kit (Qiagen, #28106) and analyzed by qPCR using the primers listed in Table S3. The IgG background was subtracted, and the datasets were normalized to input values as follows:

$$percent\;input=2^{{CT}_{input}-{CT}_{IP}}\times 25$$

ChIP DNA libraries were generated using NEB Next Ultra II DNA Library Prep Kit (NEB, #E7103) with NEBNext Multiplex Oligos for Illumina (96 Unique Dual Index Primer Pairs, #E6440S) per the manufacturer’s protocols. Sequencing was then performed by the Genomics Core of the Herbert Wertheim UF Scripps Institute for Biomedical Innovation & Technology.

### ChIP sequencing analysis

Quality control on the reads were assessed using FastQC (Braham Bioinformatics) and MultiQC.^105^ Trim_galore (-- paired --length 24 --stringency 3) was then used to trim the paired-end .fastq files. The alignment was performed on a custom human genome GRCh38 with HIV-R7/E−/GFP inserted into chromosome 9 at position 136,468,578. Then, duplicate reads were removed, and quality was filtered using samtools.^106^ Peak calling was finally performed on merged replicates vs. input using macs2 (with -q 0.01).^107^ Bedtools intersect was used to calculate the enrichment of one set of peaks within another.^108^ ChIP sequencing (ChIP-seq) data were processed using deepTools to convert .bam files to .bdg files (bamCoverage) followed by conversion to .bw (BedGraphToBigWig^109^) with normalization across samples. Visualization of the ChIP-Seq data was then performed using computeMatrix, plotHeatmap, and plotProfile tools.^110^ ChIP-seq read coverage across genomic regions was generated using pyGenomeTracks.^111^ Motif analysis was generated using Homer with the findMotifsGenome.pl command.^112^

### Protein expression and purification

Recombinant FUBP3 protein was expressed as an N-terminal glutathione (GST)-FUBP3 fusion protein using a pGEX-3T-4 vector. The expression was done in Rosetta(DE3) *Escherichia coli* cells (Millipore Sigma, #70954) in lysogeny broth (LB) medium broth at 37°C for about 5 h until its optical density 600 (OD_600_) was 0.6. The temperature was cooled down to 18°C before adding 0.4 mM of isopropyl β-D-thiogalactoside (IPTG) and incubated overnight before harvesting by centrifugation (4,000 × *g*, 15 min). Purification followed the protocol from Pierce GST protein interaction pull-down kit (Thermo Fisher, #21516) where cells were resuspended in lysis buffer (25 mM Tris-HCl [pH 7.2], 150 mM NaCl, 0.1% SDS, 1% Triton X-100, and 0.2 mM phenylmethanesulfonyl fluoride [PMSF]) and following by an incubation on ice for 30 min and centrifugation (12,000 × *g*, 5 min) to remove the insoluble part. The soluble part was then incubated with Pierce Glutathione Agarose beads at 4°C on a nutator for 30 min. The flow-through was collected by centrifugation (1,250 × *g*, 30 s) for further detection. The protein-bead hybrid was washed five times with Tris-buffered saline (TBS) buffer (25 mM Tris-HCl [pH 7.2], 150 mM NaCl, 0.05% SDS, 0.1% Triton X-100, and 0.1 mM PMSF). To elute the protein, elution buffer (5 mM glutathione, 25 mM Tris-HCl [pH 7.2], 150 mM NaCl, 0.05% SDS, 0.1% Triton X-100, and 0.1 mM [PMSF]). was added to the hybrid and incubated for 5 min on a nutator and centrifuged to obtain the eluted GST-FUBP3 protein. The eluted protein was confirmed by western blotting using both GST (Santa Cruz Biotechnology, #sc-138) and FUBP3 (Abcam, #ab181025) antibodies.

### Recombinant protein interaction assay

1 μg of His-Tat and 10 μg of GST-FUBP3 was mixed in TBS buffer and incubated at 4°C on a nutator for 10 min. 1 μg of His antibody (Invitrogen, #MA1-21315) was added to the two protein and incubated for another 1 h at 4°C. 8 μL of Dynabeads Protein G (Invitrogen, #10004D) was added after equilibrating with TBS buffer and a 1-h incubation applied. After washing the proteins-beads hybrid five times, the proteins were eluted with 1× Laemmli buffer and boiled for 10 min at 95°C. The interaction was confirmed by western blotting.

### Immunoprecipitation assay

After 48-h transfection, cells were lysed with immunoprecipitation (IP) buffer (50 mM Tris-HCl pH 7.5, 1 mM EDTA, 150 mM NaCl, 1% Triton X-100 + proteinase inhibitors [Roche, #05892791001]) for 15 min on ice, and the lysate was then centrifuged 10 min at 13,000 rpm at 4°C. Protein concentration in the supernatant was evaluated by Bradford assay (Bio-Rad, #5000006). One milligram of cell lysate was incubated with 4 μg of antibody overnight at 4°C on rotor. Then, 15 μL (per sample) of Dynabeads Protein G (Invitrogen, #10004D) were washed 2× with IP buffer, added to the samples, and incubated for 1 h at 4°C on rotor. The antibody-conjugated beads were then washed 5× with IP buffer, resuspended in 40 μL of 1× Laemmli buffer, and boiled for 10 min at 95°C followed by western blot.

### RNA/DNA pull-down assay

Based on previous protocol,^88^ 5 μL of streptavidin beads were washed 2× with 1 mL of binding buffer (20 mM Tris-HCl pH 7.5, 2.5 mM MgCl_2_, 100 mM NaCl) and blocked with 16 μg of tRNA (Invitrogen, #AM119) and 25 μg of BSA (Sigma-Aldrich, A2153) for 30 min at 4°C. Then, 1 μg of biotin-labeled RNA or DNA oligomer (*TAR*, *TAR*-Del, *TAR*-mut; see Table S1) was added to the beads and incubated for 30 min at 4°C. The oligo-bead complex was washed 2× with 1 mL of binding buffer and the beads were resuspended in 250 μL of resuspension buffer buffer (50 mM Tris-HCl pH 8.0, 5 mM MgCl_2_, 5 mM MnCl_2_, 10 μM ZnCl_2_, 1 mM DTT, 100 mM NaCl). Then, the beads were added to 250 μL of cell lysates (250 μg) prepared from 3 × 10^6^ HEK293T cells with cell lysis buffer (50 mM Tris-HCl pH 7.5; 1 mM EDTA; 150 mM NaCl; 1% Triton X-100 + RNase, DNase, and proteinase inhibitors) and incubated for 2 h at 4°C. Samples were washed 5× with cell lysis buffer, resuspended in 40 μL of 1× Laemmli buffer, and boiled for 10 min at 95°C followed by western blot.

### Tat degradation assay

In a six-well plate, HEK293T cells were transfected with 2.5 μg of either pMKO.1-puro shFUBP3 or shCD8B for 24 h. The next day, cells were transfected with 0.5 μg of Tat-FLAG-WT or Tat-FLAG-BRM for 24 h. The next day, cells were treated with 100 μg/mL of CHX and lysed at different time points (0, 1, 2, and 4 h) followed by western blot analysis.

### RIP assay

RIP was performed using the EZ-Magna RIP Kit (Millipore, #17-701) per the manufacturer’s recommended protocol. Briefly, cell lysates were prepared with 3 × 10^7^ Jurkat-D6 cells, stimulated overnight with 10 ng/mL of TNF-α (Sigma-Aldrich #T6674), and lysed with RIP lysis buffer supplemented with RNase and protease inhibitors. Lysate samples were then incubated overnight at 4°C with antibody-coated beads (Abcam, #ab181025). After a total of six washes with ice-cold RIP wash buffer, the bead-bound immunocomplexes were digested with proteinase K at 55°C for 30 min. Using a magnetic separator, supernatants were used to extract RNA by phenol/chloroform extraction followed by ethanol precipitation before qPCR or RNA-seq analysis as previously described. The raw .fastq files were aligned to a made-in-house human genome (Hg38) supplemented with the HIV gene using STAR.^98^ Gene expression changes were estimated with Featurecounts^99^ and Deseq2.^100^ The fgsea package was used to conduct gene pathway analysis.^82^^,^^102^^,^^103^^,^^104^

### *TAR*-RNA degradation assay

J-Lat 10.6 cells were transduced with pMKO-puro vector packaged with pUMVC3 (Addgene #8449) and pCVM-VSV-G (Addgene #8454) expressing shCD8 or shFUBP3, followed by selection with puromycin (1.5 μg/mL) for 7 days. In a 24-well plate, 1 million cells were treated with 100 nM flavopiridol, and cells were collected at different time points (0, 2, 4, 8, and 24 h) followed by RNA extraction and RT-qPCR analysis as previously described.

## Data availability

The supplemental information includes Figures S1–S19, Tables S1–S3, and experimental methods. The results of RNA-seq, ChIP-seq, and RIP-seq analysis were deposited in Mendeley Data (https://doi.org/10.17632/wvkhpsbbfs.1).

## Acknowledgments

This work was supported by National Institute of Allergy and Infectious Disease, NIAID (R01AI097012, R37AI165137, UM1AI164559, R01AI177327, R33 AI140439, R21AI158296, and R01AI167732). Schematics were created with BioRender.com.

## Author contributions

Conceptualization, Q.M.R.G., C.L., and S.T.V.; formal analysis, Q.M.R.G; resources, C.L., A.C., I.M., L.P.M., and S.T.V.; data curation, Q.M.R.G.; writing – original draft, Q.M.R.G. and S.T.V.; writing – review & editing, Q.M.R.G., C.L., A.C., and S.T.V.; visualization, Q.M.R.G.; investigation, Q.M.R.G., C.L., A.C., I.M., and L.P.M.; methodology, Q.M.R.G., C.L., and S.T.V.; software, Q.M.R.G; project administration, S.V., Q.M.R.G., and C.L.; supervision, S.T.V.; funding acquisition, S.T.V.

## Declaration of interests

The authors declare no competing interests.

## Supplemental information

Document S1. Figures S1–S19 and Tables S1–S3

Document S2. Article plus supplemental information
