# FUBP3 enhances HIV-1 transcriptional activity and regulates immune response pathways in T cells

**DOI:** 10.1016/j.omtn.2025.102544

**Published:** 2025-04-24

**Authors:** Quentin M.R. Gibaut, Chuan Li, Anqi Cheng, Ines Moranguinho, Luisa P. Mori, Susana T. Valente

## Main text

(Molecular Therapy: Nucleic Acids *36*; June 2025)

In the originally published version of this article, Figure 5E displayed the wrong western blot of the loading control H3. The blot was updated with the right image to correct that mistake. The authors sincerely apologize for any inconvenience this may have caused.

**Figure:**
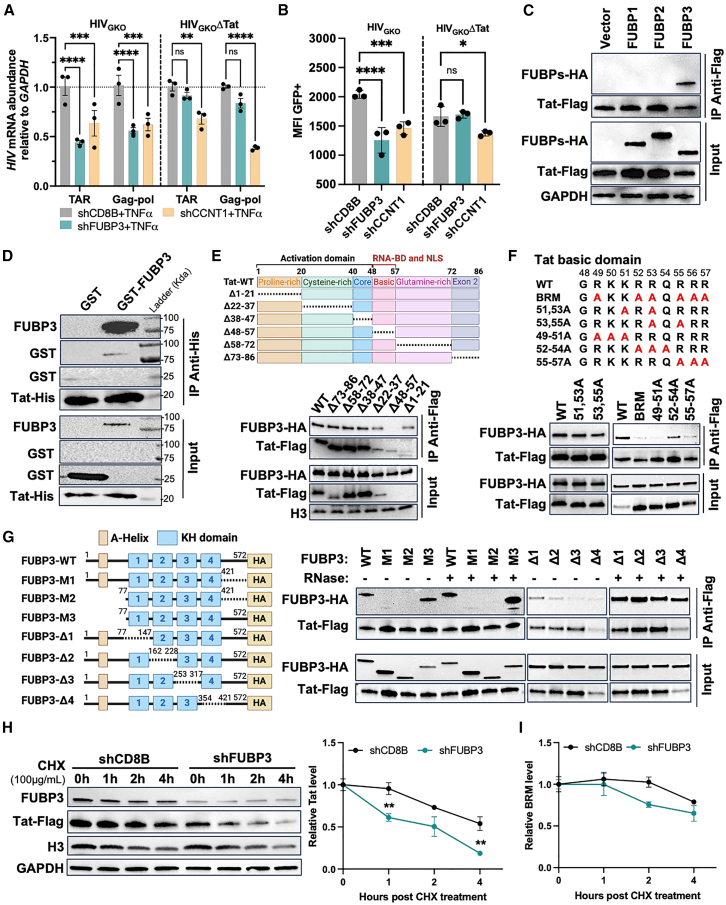
Figure 5. FUBP3 regulates HIV-1 in a Tat-dependent manner (original)

**Figure:**
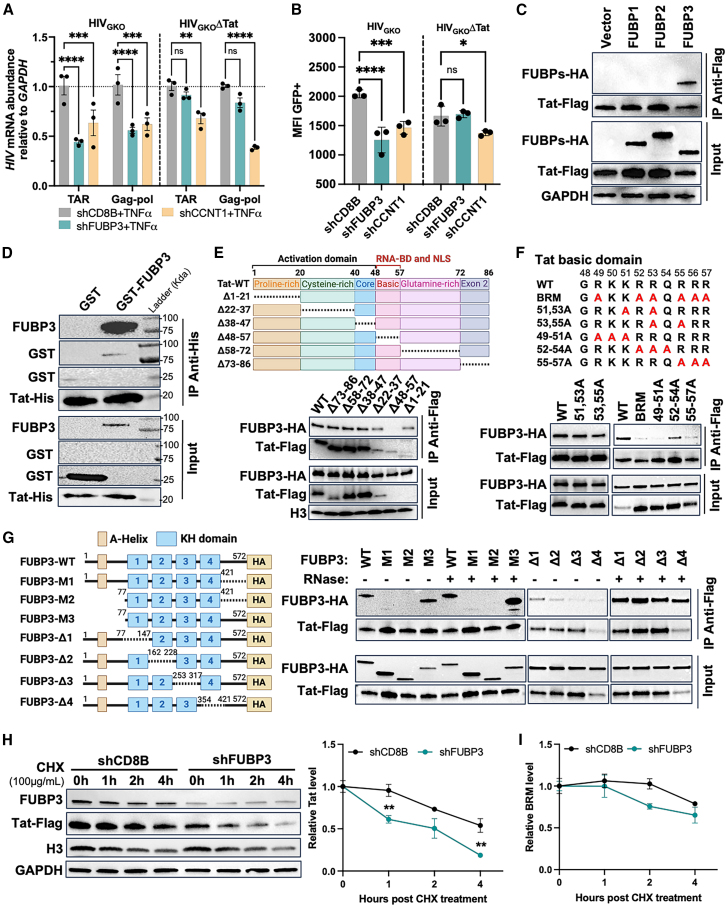
Figure 5. FUBP3 regulates HIV-1 in a Tat-dependent manner (corrected)

