# Supplementary material for: FUBP3 enhances HIV-1 transcriptional activity and regulates immune response pathways in T cells
Source: Mol Ther Nucleic Acids. 2025 Mar 25;36(2):102525. doi: 10.1016/j.omtn.2025.102525 (PMC12005928; doi:10.1016/j.omtn.2025.102525)
Supplement: Document S1. Figures S1–S19 and Tables S1–S3 [file mmc1.pdf]

OMTN, Volume *36*

Supplemental information

FUBP3 enhances HIV-1 transcriptional activity and regulates immune response pathways in T cells

Quentin M.R. Gibaut, Chuan Li, Anqi Cheng, Ines Moranguinho, Luisa P. Mori, and Susana T. Valente

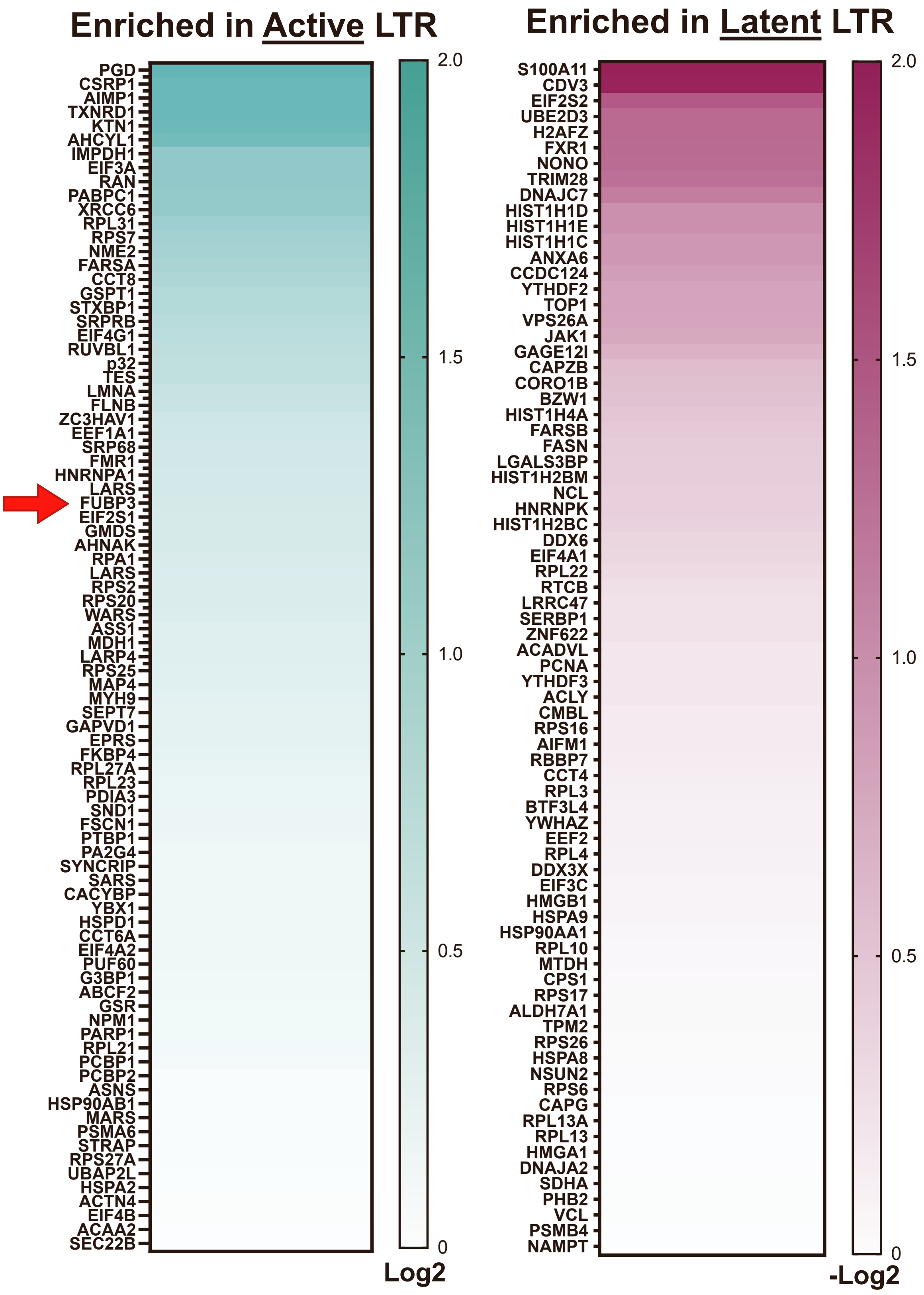

Enriched in Active LTR
PGD
CSRP1
AIMP1
TXNRD1
KTN1
AHCYL1
IMPDH1
EIF3A
RAN
PABPC1
XRCC6
RPL31
RPS7
NME2
FARSA
CCT8
GSPT1
STXBP1
SRPRB
EIF4G1
RUVBL1
p32
TES
LMNA
FLNB
ZC3HAV1
EEF1A1
SRP68
FMR1
HNRNPA1
LARS
FUBP3
EIF2S1
GMDS
AHNAK
RPA1
LARS
RPS2
RPS20
WARS
ASS1
MDH1
LARP4
RPS25
MAP4
MYH9
SEPT7
GAPVD1
EPRS
FKBP4
RPL27A
RPL23
PDIA3
SND1
FSCN1
PTBP1
PA2G4
SYNCRIP
SARS
CACYBP
YBX1
HSPD1
CCT6A
EIF4A2
PUF60
G3BP1
ABCF2
GSR
NPM1
PARP1
RPL21
PCBP1
PCBP2
ASNS
HSP90AB1
MARS
PSMA6
STRAP
RPS27A
UBAP2L
HSPA2
ACTN4
EIF4B
ACAA2
SEC22B
2.0
1.5
1.0
0.5
0
Log2
Enriched in Latent LTR
S100A11
CDV3
EIF2S2
UBE2D3
H2AFZ
FXR1
NONO
TRIM28
DNAJC7
HIST1H1D
HIST1H1E
HIST1H1C
ANXA6
CCDC124
YTHDF2
TOP1
VPS26A
JAK1
GAGE12I
CAPZB
CORO1B
BZW1
HIST1H4A
FARSB
FASN
LGALS3BP
HIST1H2BM
NCL
HNRNPK
HIST1H2BC
DDX6
EIF4A1
RPL22
RTCB
LRRC47
SERBP1
ZNF622
ACADVL
PCNA
YTHDF3
ACLY
CMBL
RPS16
AIFM1
RBBP7
CCT4
RPL3
BTF3L4
YWHAZ
EEF2
RPL4
DDX3X
EIF3C
HMGB1
HSPA9
HSP90AA1
RPL10
MTDH
CPS1
RPS17
ALDH7A1
TPM2
RPS26
HSPA8
NSUN2
RPS6
CAPG
RPL13A
RPL13
HMGA1
DNAJA2
SDHA
PHB2
VCL
PSMB4
NAMPT
2.0
1.5
1.0
0.5
0
-Log2

Figure S1. ChAP-MS identifies FUBP3 enriched on active HIV-1 promoter loci. Previously reported[1] results from ChAP-MS in HeLa-M1. NL4-3 chronically infected HeLa-M1 cells expressing dCAS9 and gRNAs were treated with ART+DMSO (Active LRT, heatmap on the left) or ART+dCA (100 nM, Latent LTR, heatmap on the right) and crosslinked with formaldehyde. Chromatins were sheared by sonication, immunoprecipitated, purified and samples were analyzed by mass spectrometry. The scale represents Log2 (Active LTR) and -Log2 (Latent LTR) of the enrichment ratio DMSO/dCA. FUBP3 was found enriched in active LTR (red arrow).

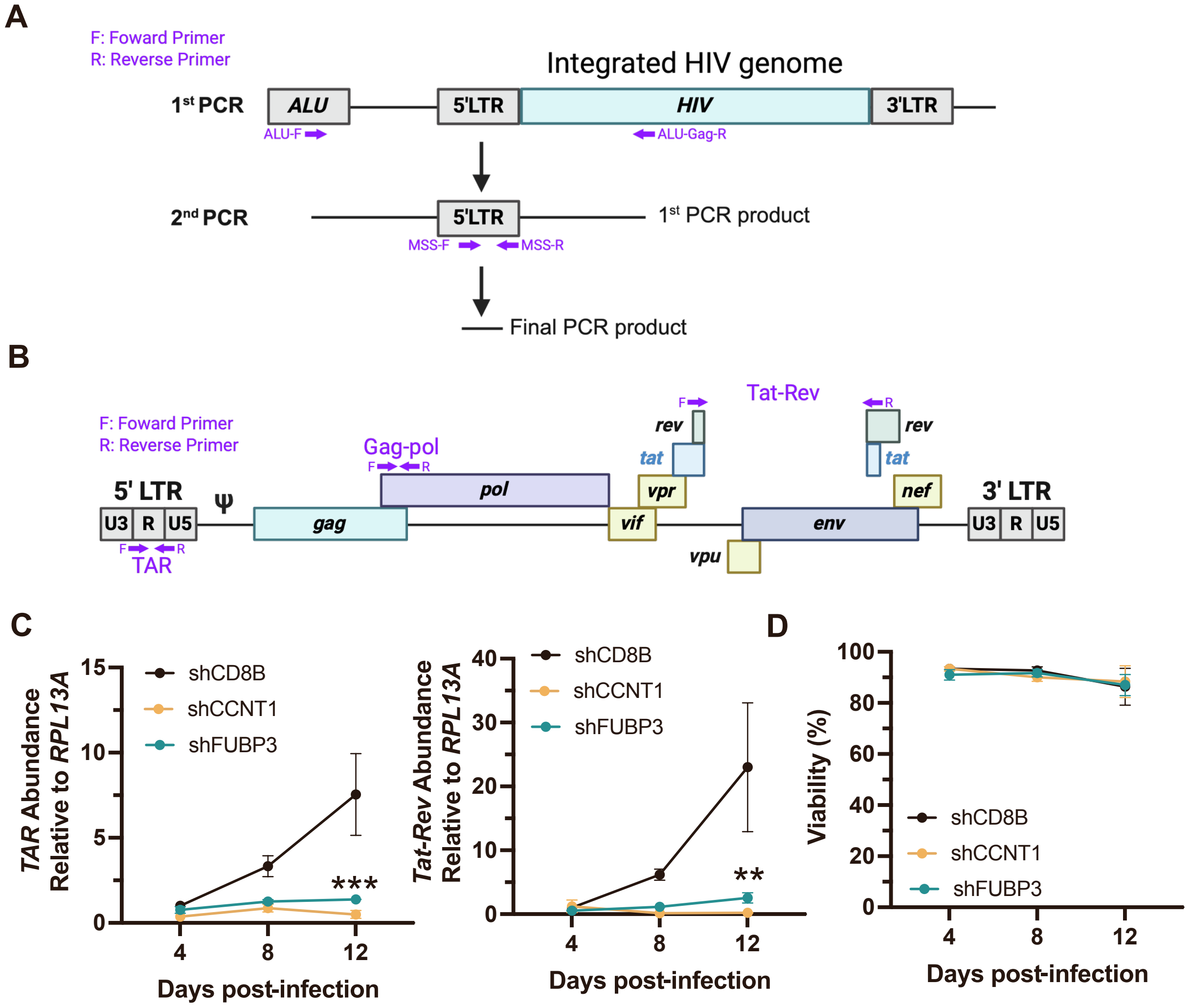

Figure S2. FUBP3 is essential for HIV transcription and replication in infected Jurkat CD4⁺T cells. (A) Schematic of primers location for the HIV DNA integration assay. (B) Schematic of primers location on the HIV genome used for RT-qPCR. (C) Effect of FUBP3 and CCNT1 depletion on *TAR* and *Tat-Rev* abundance in infected Jurkat CD4$^+$T cells overtime post infection. (D) Cell viability of Jurkat CD4$^+$T cells overtime post-infection as determined by trypan blue staining. **, $p < 0.01$; ***, $p < 0.001$; as determined by two-way ANOVA with multiple comparisons. All data are reported as the mean ± SEM.

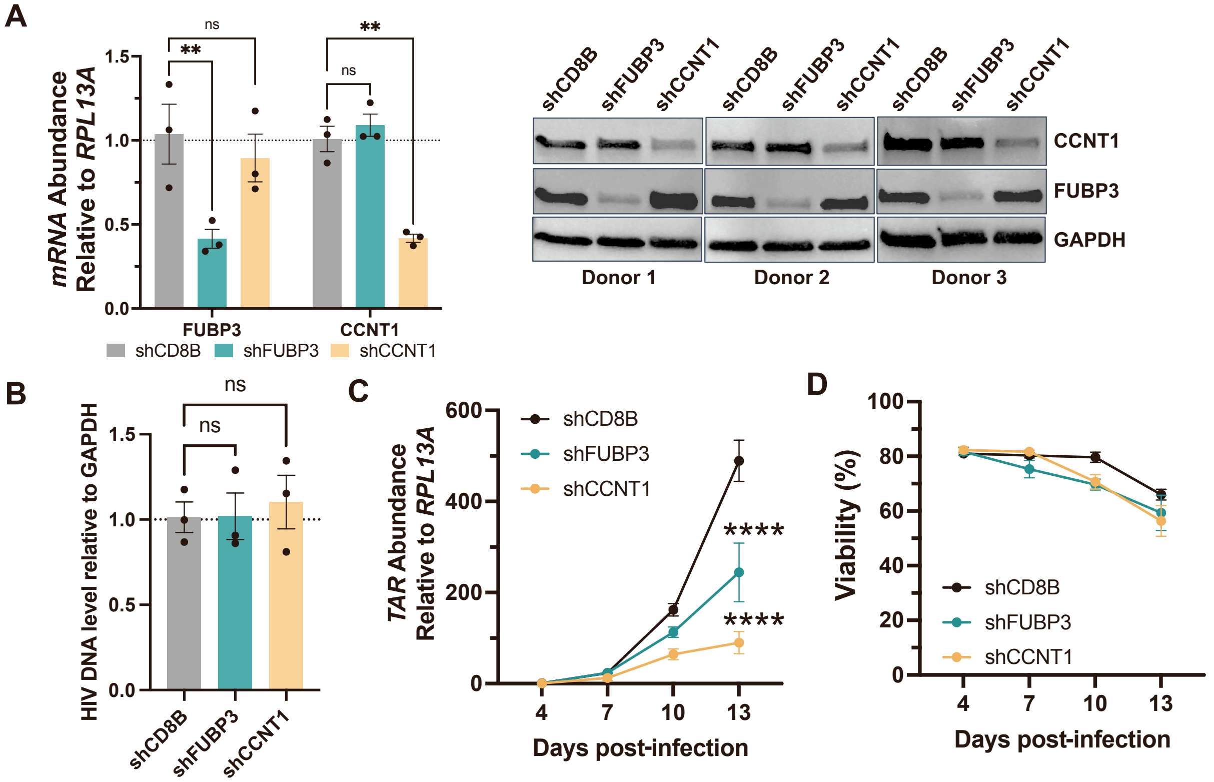

Figure S3. Knock-down by shRNAmirs and HIV infection in primary CD4⁺T cells. (A) Knock-down efficiency for FUBP3 and CCNT1 monitored by RT-qPCR and western blot in primary CD4⁺T cells from 3 donors. (B) HIV integration in primary CD4⁺T cells quantified by ALU-PCR assay. (C) Effect of FUBP3 and CCNT1 depletion on *TAR* abundance in infected primary CD4⁺T cells overtime post-infection. (D) Cell viability of primary CD4⁺T cells overtime post-infection as determined by trypan blue staining. **, $p < 0.01$; ****, $p < 0.0001$; as determined by two-way ANOVA with multiple comparisons. All data are reported as the mean ± SEM.

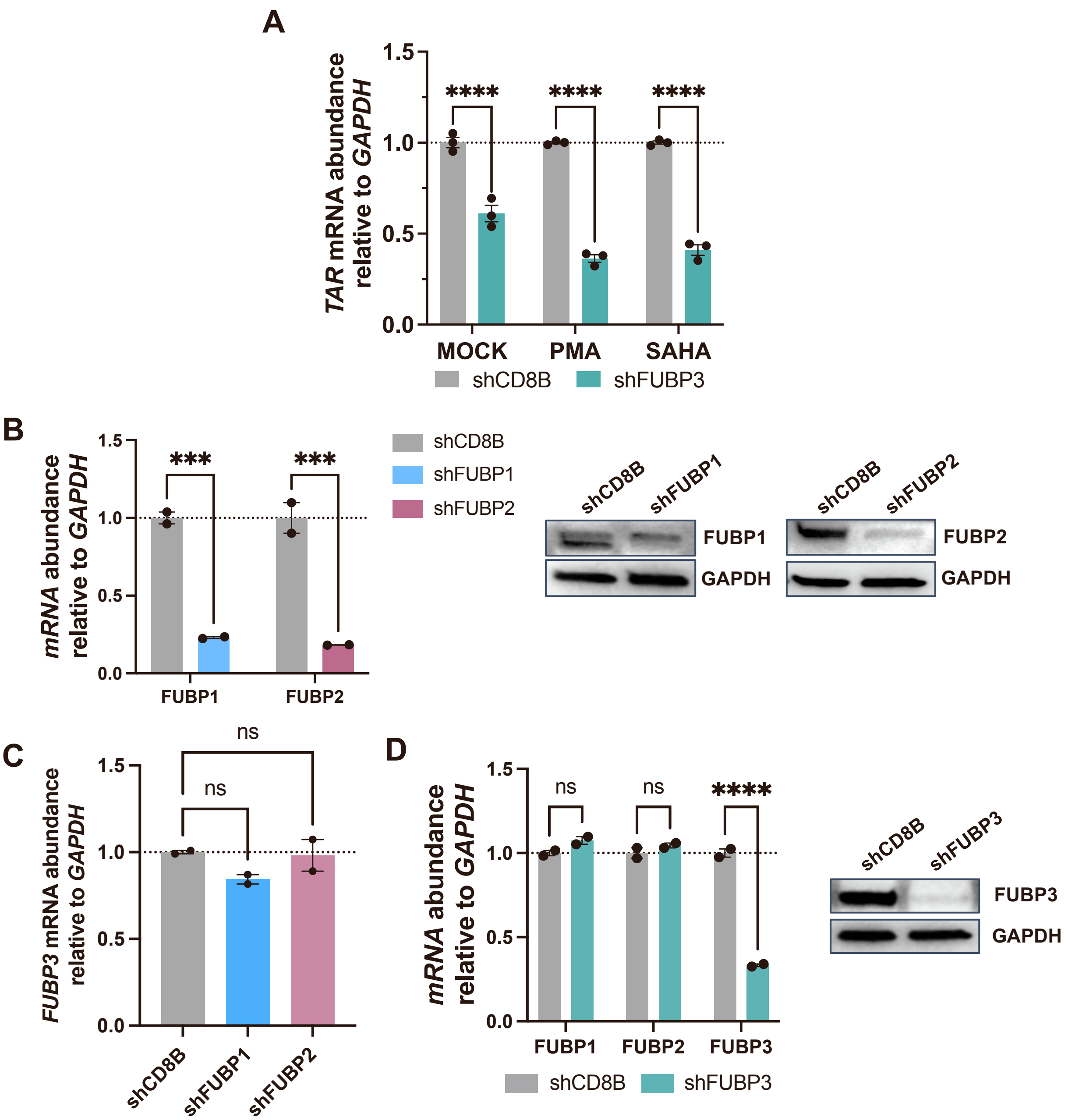

Figure S4. Effect of FUBP3 depletion in Jurkat-D6 cells. (A) *TAR* transcript abundance upon FUBP3 depletion with or without stimulation (MOCK, PMA, SAHA) in Jurkat-D6 cells as determined by RT-qPCR. (B) Knock-down efficiency for *FUBP1* and *FUBP2* monitored by RT-qPCR (left) and western blot (right) in Jurkat-D6 cells. (C) *FUBP3* mRNA abundance upon knock-down with shFUBP1 and shFUBP2 determined by RT-qPCR. (D) *FUBP1*, *FUBP2* and *FUBP3* mRNA abundance upon knock-down with shFUBP3 determined by RT-qPCR (left) and knock-down efficiency monitored by western blot (right). ***, $p < 0.001$; ****, $p < 0.0001$; as determined by two-way ANOVA with multiple comparisons. All data are reported as the mean ± SEM.

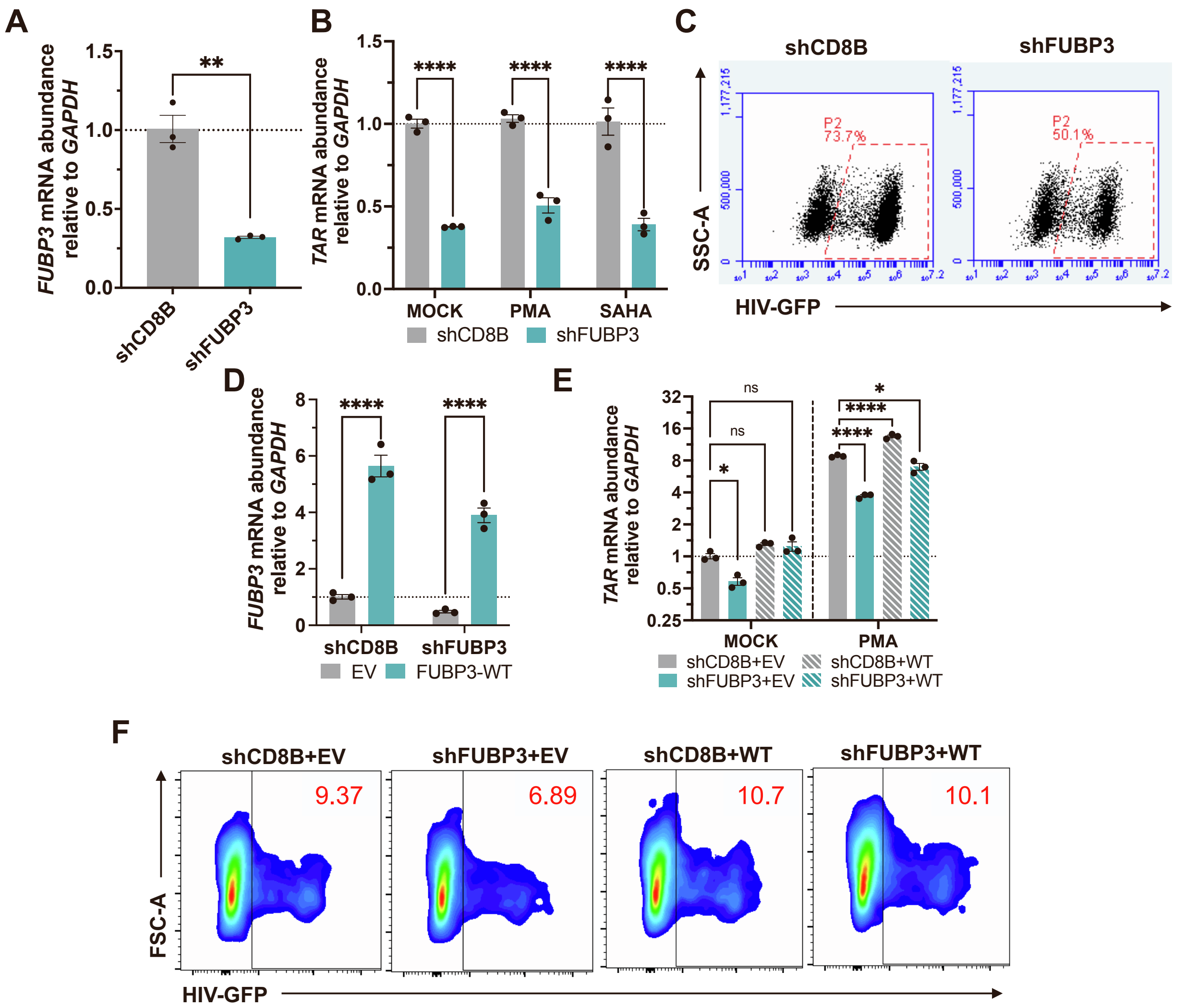

Figure S5. FUBP3 activates HIV transcription in J-Lat 10.6 cells. (A) Expression of *FUBP3* in J-Lat 10.6 cells transduced with shRNAmirs targeting CD8B or FUBP3 as determined by RT-qPCR. (B) *TAR* transcript abundance upon FUBP3 depletion with or without stimulation (MOCK, PMA, SAHA) in J-Lat 10.6 cells. (C) Representative flow cytometry plot (from Fig. 2F) showing expression of HIV(GFP%). (D-E) Expression of *FUBP3* and *TAR* in J-Lat 10.6 cells (from Fig. 2G) as determined by RT-qPCR. (F) Representative flow cytometry plot (from Fig. 2I) showing expression of HIV(%GFP). *, $p < 0.05$; **, $p < 0.01$; ****, $p < 0.0001$; as determined by student T-test or two-way ANOVA with multiple comparisons. All data are reported as the mean ± SEM.

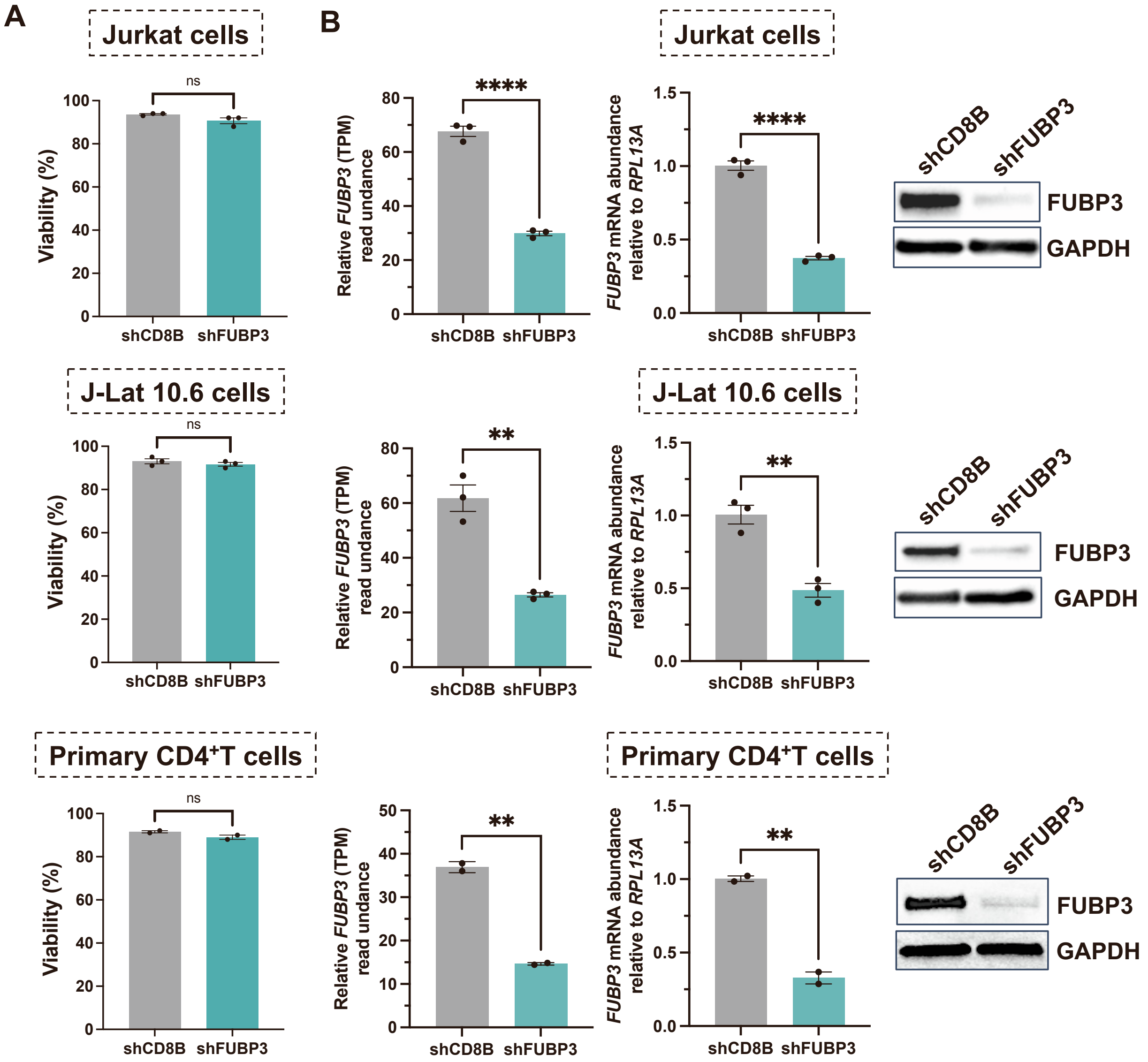

Figure S6. RNA-sequencing validation upon FUBP3 depletion. (A) Cell viability in Jurkat, J-Lat 10.6 and primary CD4^{+}T cells as determined by trypan blue staining. (B) FUBP3 depletion validation by RNA-sequencing (left), RT-qPCR (middle) and western blot (right) in Jurkat, J-Lat 10.6 and primary CD4^{+}T cells. **, $p < 0.01$; ****, $p < 0.0001$; as determined by student T-test. All data are reported as the mean ± SEM.

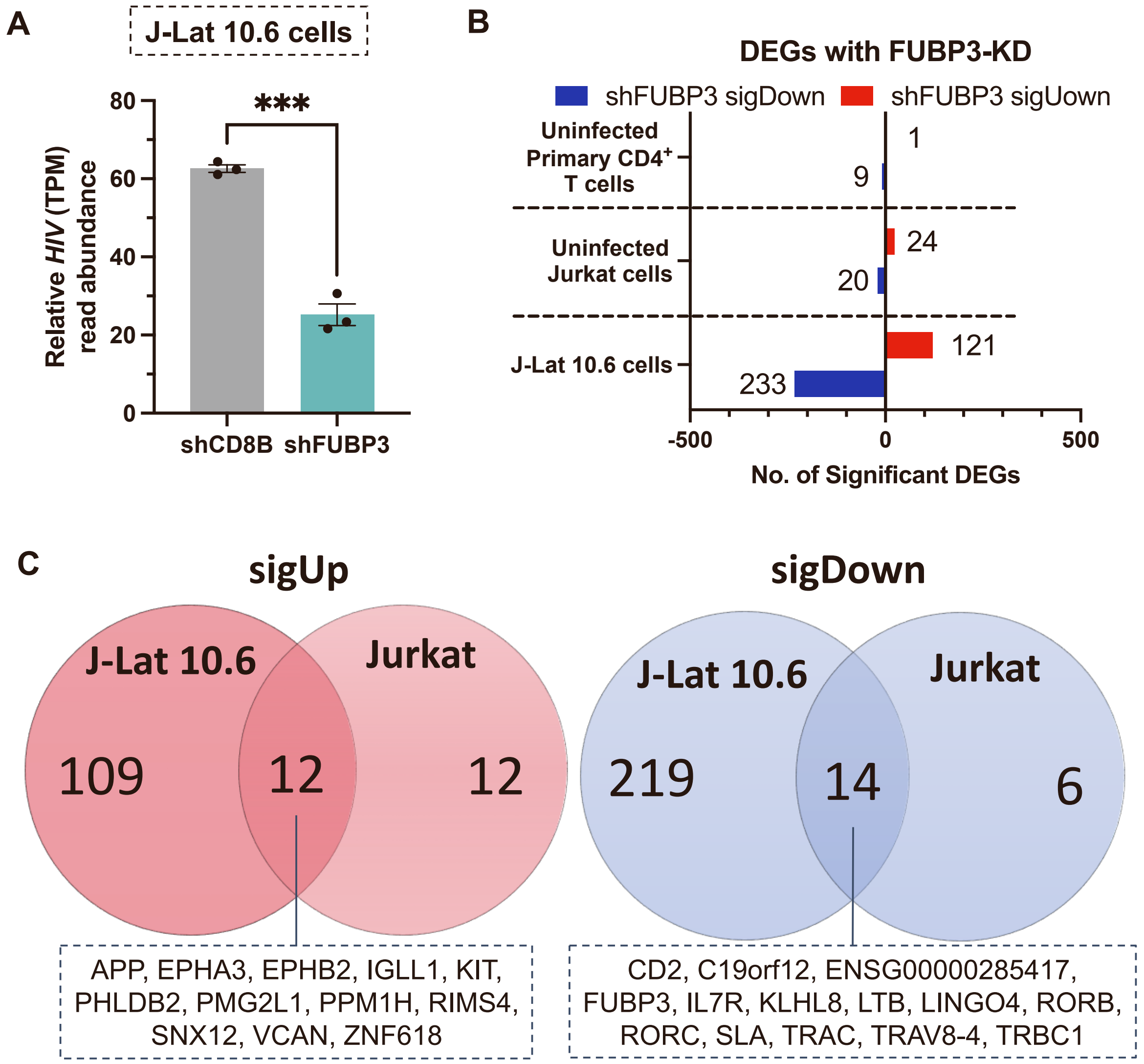

Figure S7. RNA-sequencing analysis upon FUBP3 depletion. (A) HIV-1 transcripts per million (TPM) RNA-seq reads in J-Lat 10.6 cells upon depletion with FUBP3. (B) The number of significant DEGs in each cell model with FUBP3-KD. (C) Venn diagram showing common dysregulated genes (Up in red, down in blue) in Jurkat and J-Lat 10.6 cells. ***, $p < 0.001$; as determined by student T-test. All data are reported as the mean ± SEM.

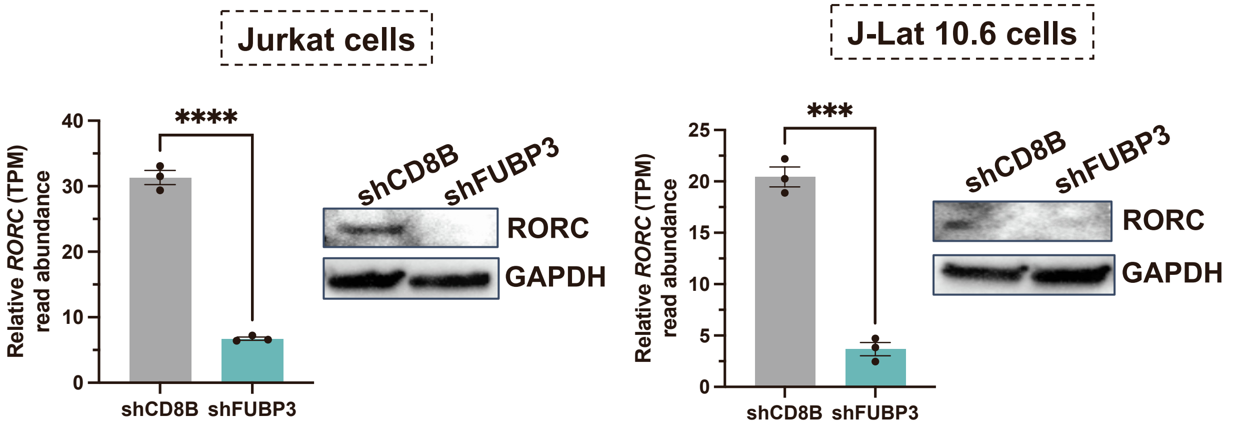

Figure S8. Transcriptional effect of FUBP3 depletion on RORC in Jurkat and J-Lat 10.6 cells. Transcripts per million (TPM) RNA-seq reads of *RORC* in Jurkat and J-Lat 10.6 cells upon depletion with FUBP3 and their respective validation by western blot. ***, $p < 0.001$; ****, $p < 0.0001$; as determined by student T-test. All data are reported as the mean ± SEM.

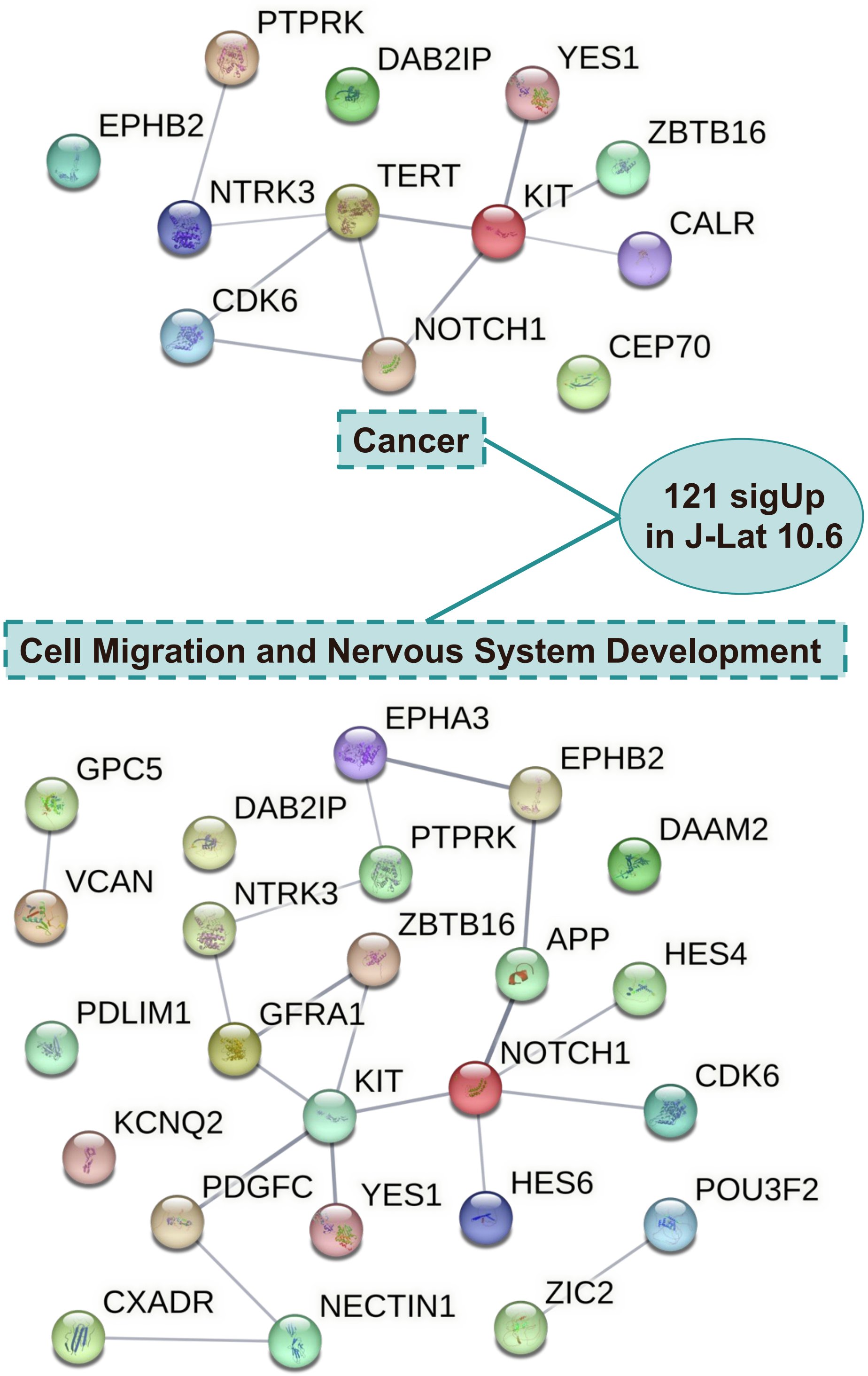

Figure S9. RNA-Seq network pathway analysis of upregulated genes in J-Lat 10.6 cells. Protein network of upregulated genes in J-Lat 10.6 cells upon FUBP3 depletion grouped by function using STRING db v12. Each node represents a protein, and line thickness indicate confidence in both functional and physical protein associations.

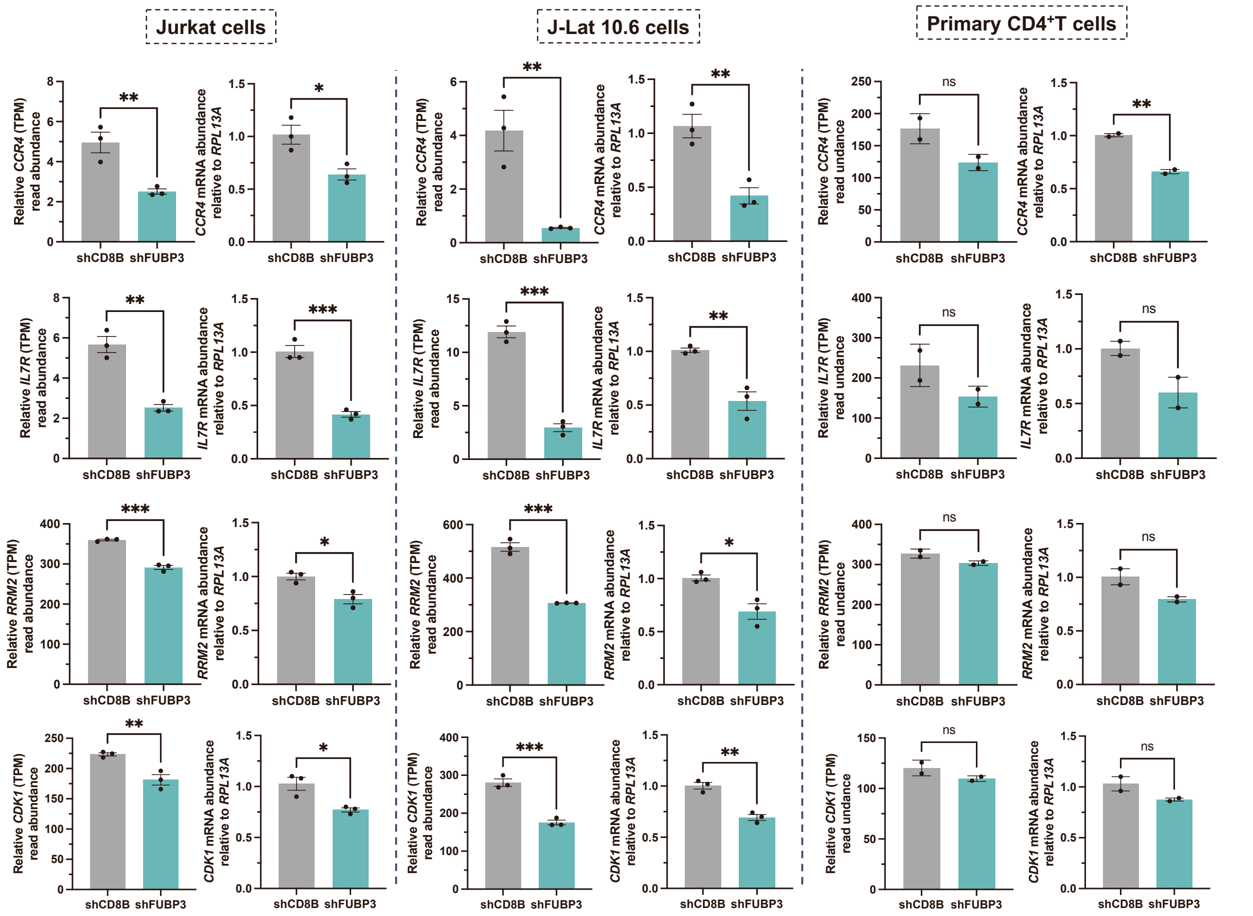

Figure S10. Transcriptional effect of FUBP3 depletion in CD4+T cells. Transcripts per million (TPM) RNA-seq reads in Jurkat, J-Lat 10.6 and primary CD4+T cells upon depletion with FUBP3 and their respective validation by RT-qPCR of *CCR4*, *IL7R*, *RRM2* and *CDK1*. *, $p < 0.05$; **, $p < 0.01$; ***, $p < 0.001$; as determined by student T-test. All data are reported as the mean ± SEM.

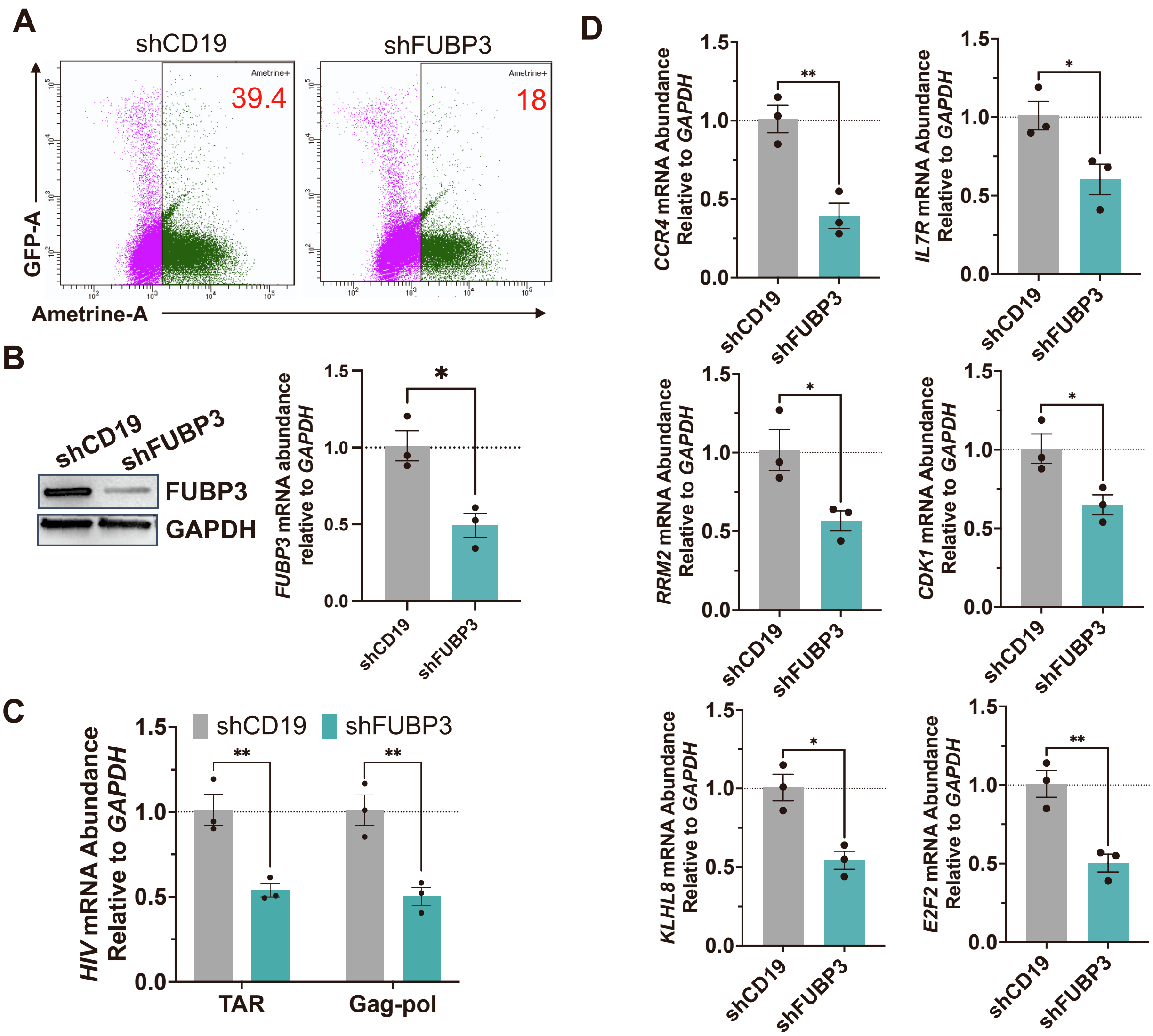

Figure S11. Validation of downregulated genes in J-Lat 10.6 cells using LMPd-Ametrine shRNA vector. (A) Representative flow cytometry plots 6 days post transduction and prior to cell sorting. All ametrine positive cells were sorted and cultured for the rest of the assay. (B) shRNAmirs depletion of FUBP3 and control CD19 in J-Lat 10.6 cells by western blot and RT-qPCR post cell sorting. (C) *TAR* and *Gag-pol* transcript abundance upon FUBP3 depletion in J-Lat 10.6 cells. (D) Validation by RT-qPCR of *CCR4*, *IL7R*, *RRM2, CDK1, KLHL8* and *E2F2* in J-Lat 10.6 cells where FUBP3 was depleted with LMPd-Ametrine shRNA vector. *, $p < 0.05$; **, $p < 0.01$; as determined by student T-test. All data are reported as the mean ± SEM.

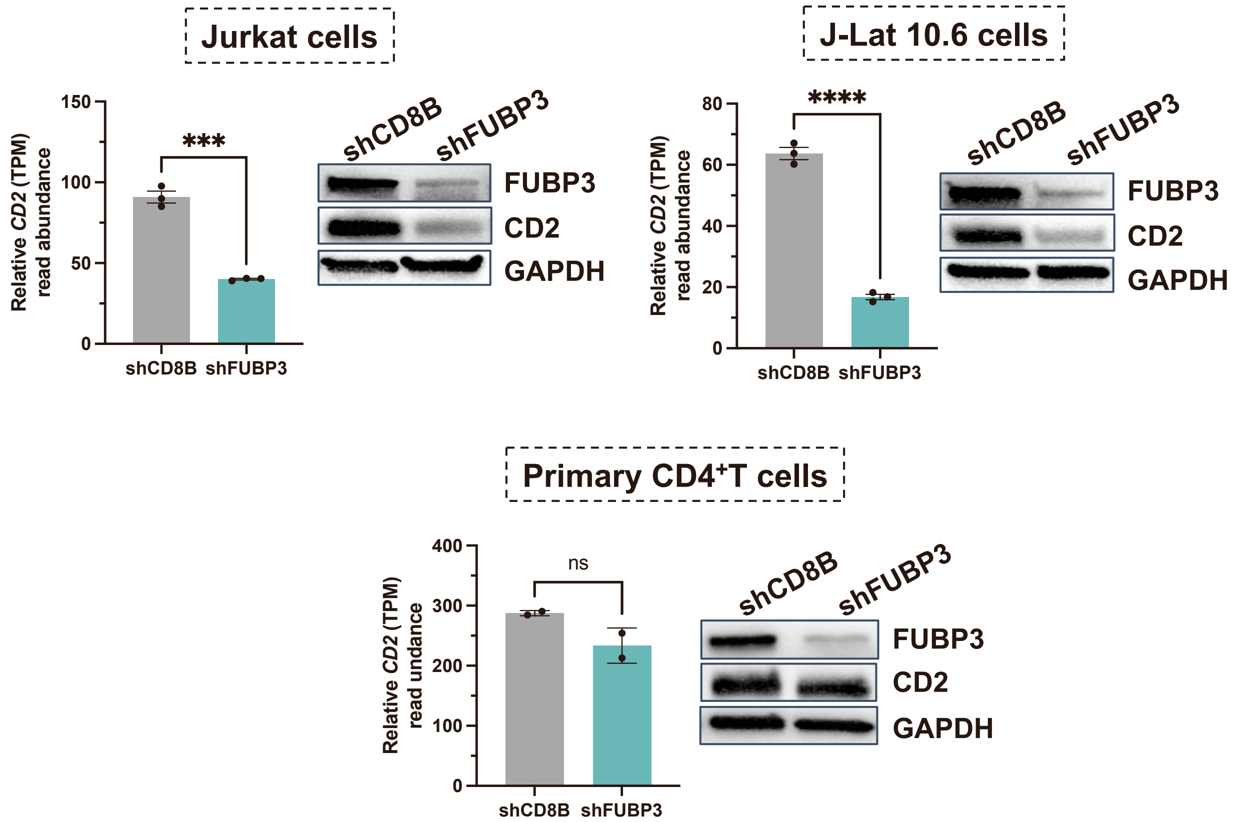

Figure S12. FUBP3 depletion inhibits CD2 expression in CD4⁺T cells. Dor each cellular model: (Left) CD2 transcripts per million (TPM) RNA-seq reads in Jurkat, J-Lat 10.6 and primary CD4⁺T cells upon depletion with FUBP3; (Right) respective protein expression by western blot. ***, $p < 0.001$; ****, $p < 0.0001$; as determined by student T-test. All data are reported as the mean ± SEM.

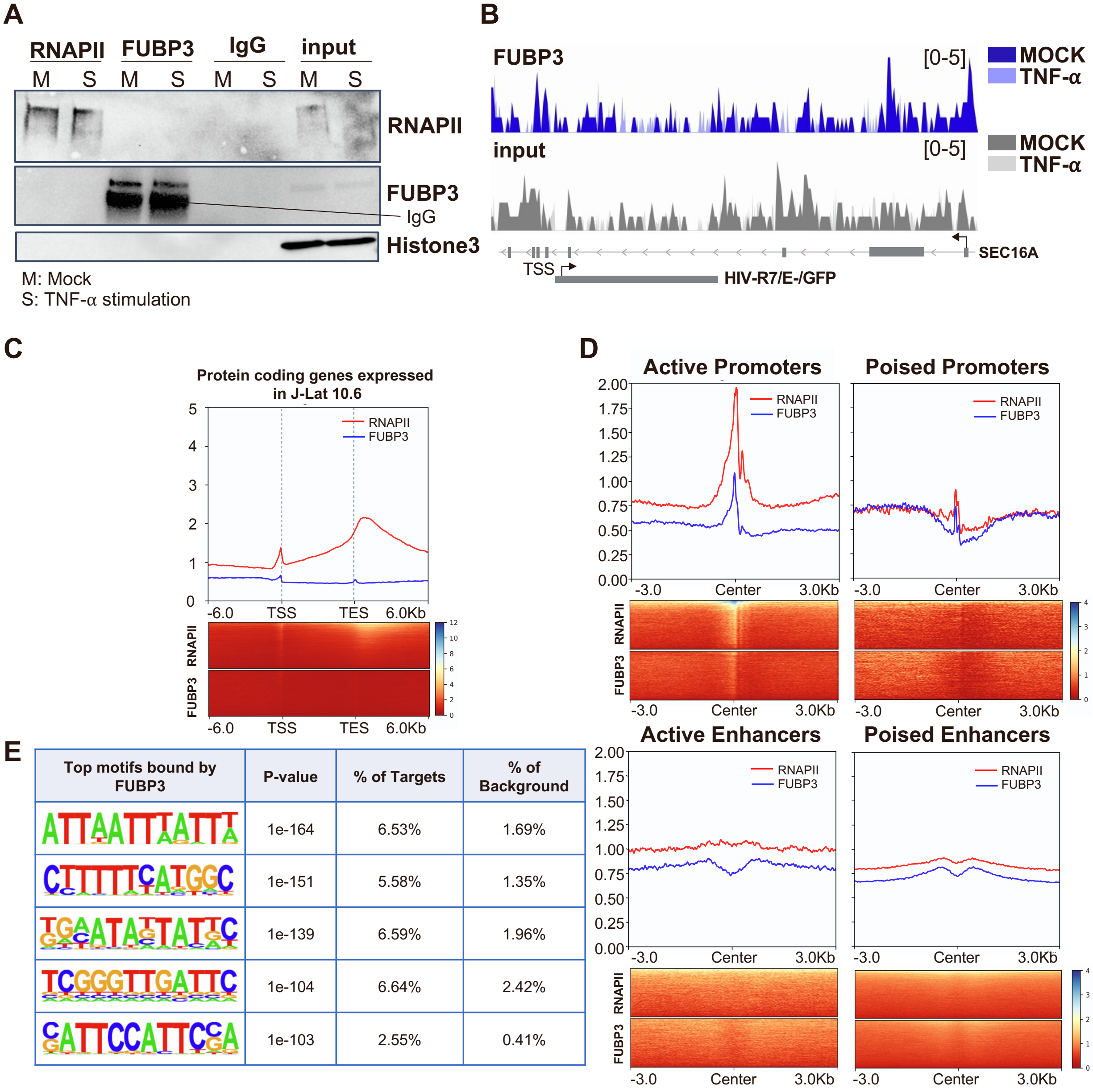

E

Top motifs bound by FUBP3	P-value	% of Targets	% of Background
ATTAATTTATTT	1e-164	6.53%	1.69%
CTTTTTCATGGC	1e-151	5.58%	1.35%
TGAATATTATTC	1e-139	6.59%	1.96%
TCGGGTTGATTC	1e-104	6.64%	2.42%
CATTCCATTCCA	1e-103	2.55%	0.41%

Figure S13. Genome wide distribution of RNAPII and FUBP3. (A) Representative image of western blot depicting the pull-down of the indicated factor following native-ChIP. (B) Genome tracks of native ChIP-seq of FUBP3 and input in J-Lat 10.6 cells with or without stimulation (MOCK or TNF-α) zoomed in to [0-5]. (C) Scaled average normalized coverage of indicated factors at all protein coding genes expressed in J-Lat 10.6 cells (15,020 genes). (D) Normalized average ChIP-Seq coverage of indicated factors across scaled genomic regions of active promoters, poised promoters, active enhancers and poised enhancers. (E) Top motifs bound by FUBP3 (9,156 peaks) analyzed by Homer.

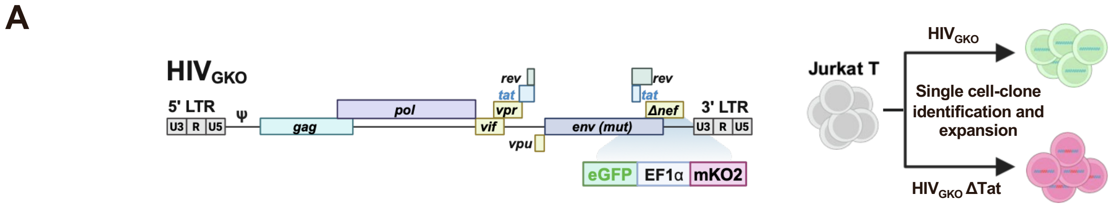

Clone name	Chromosome	Position
HIV_{GKO}	Ch 16	18548273
HIV_{GKO}ΔTat	Ch 19	10877034

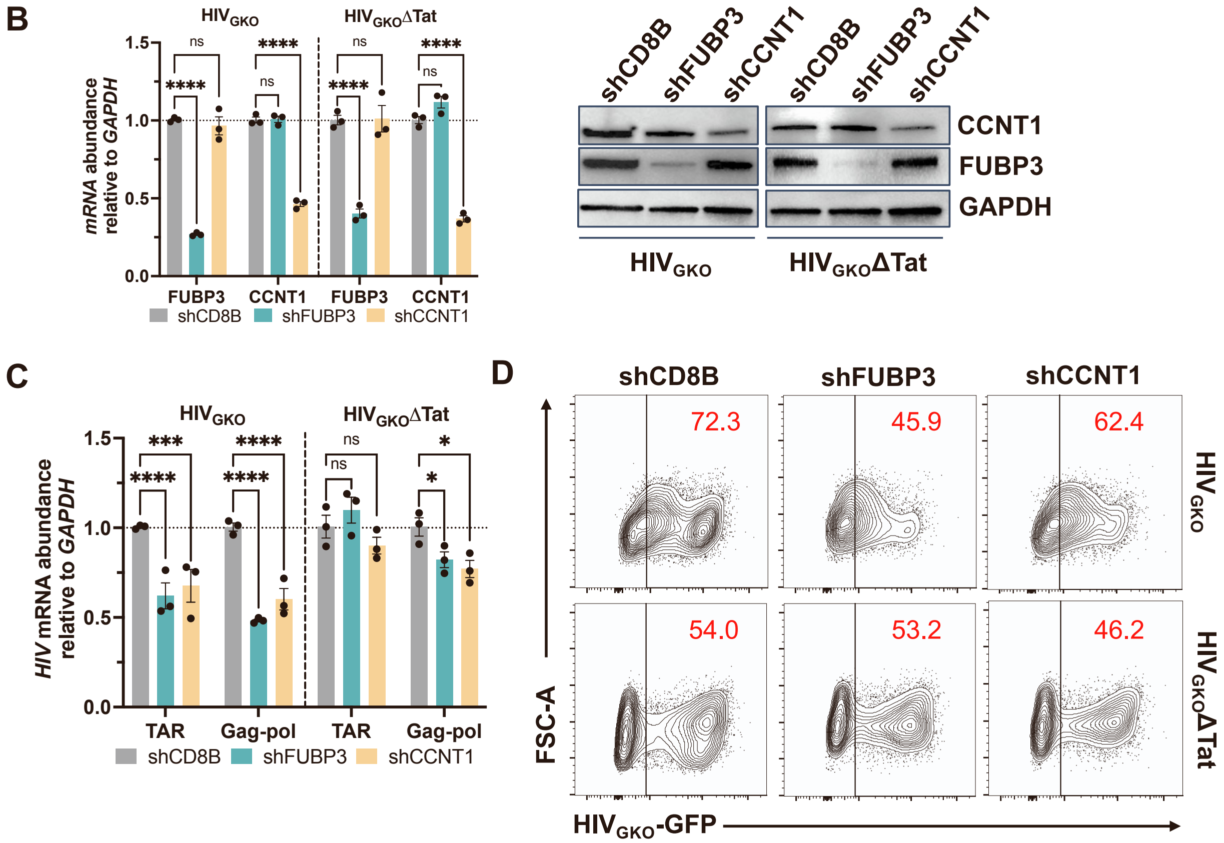

Figure S14. FUBP3 activates HIV transcription in a Tat-dependent manner. (A) The dual-fluorescence vector HIV_{GKO} with Tat depletion in Jurkat cells. (B) Knock-down efficiency for *FUBP3* and *CCNT1* monitored by RT-qPCR (left) and western blot (right) in HIV_{GKO}/HIV_{GKO}ΔTat clone cells. (C) *HIV* mRNA abundance upon FUBP3 or CCNT1 depletion in HIV_{GKO}/HIV_{GKO}ΔTat clone cells as determined by RT-qPCR. (D) Representative flow cytometry plot (from Fig. 4B) showing expression of HIV(%GFP) upon stimulation with TNF-α. **, $p < 0.01$; ***, $p < 0.001$; ****, $p < 0.0001$; as determined by two-way ANOVA with multiple comparisons. All data are reported as the mean ± SEM.

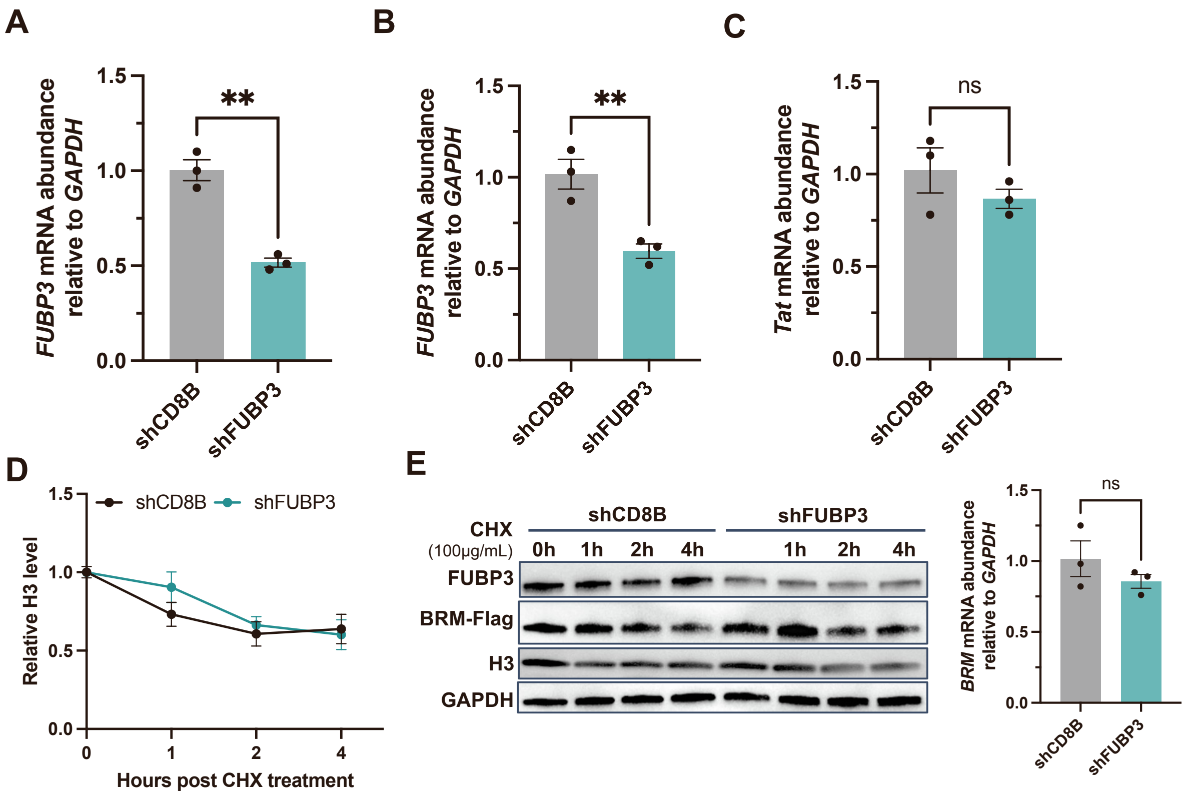

Figure S15. Experimental controls for Tat protein stabilization assay. (A) *FUBP3* mRNA level in HEK293T cells prior to Tat-Flag transfection. (B) *FUBP3* mRNA level in HEK293T cells prior to BRM-Flag transfection. (C) *Tat* mRNA level upon FUBP3 depletion as determined by RT-qPCR. (D) Histone H3 protein level quantification overtime by WB (from Fig. 5H). (D) Tat-BRM-Flag mutant degradation with FUBP3 or CD8B deletion. HEK293T cells were transfected with shCD8B or shFUBP3 followed by Tat-BRM-Flag mutant and then treated with cycloheximide (CHX) to block protein synthesis. BRM protein and mRNA level were quantified respectively by WB and RT-qPCR. **, $p < 0.01$; as determined by student T-test. All data are reported as the mean ± SEM.

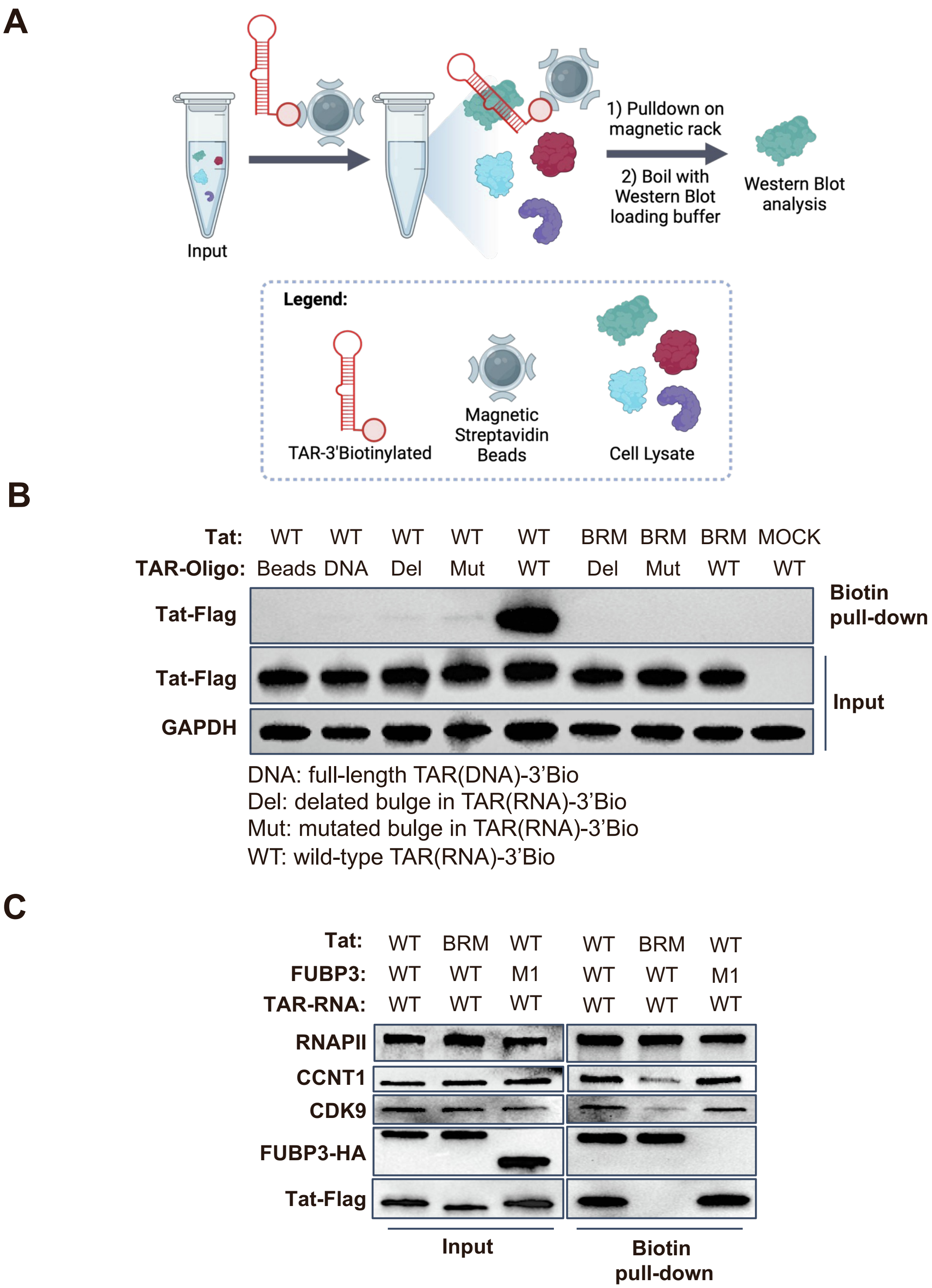

Figure S16. FUBP3 does not compete with Tat for *TAR-RNA* binding. (A) Schematic of the *in vitro TAR-RNA* pull-down assay. (B) Immunoblotting of the *in vitro TAR-RNA* pull-down with Tat. (C) Immunoblotting of the *in vitro TAR-RNA* pull-down with Tat-Flag and FUBP3-HA including a negative-binder variant (BRM for Tat and M1 for FUBP3).

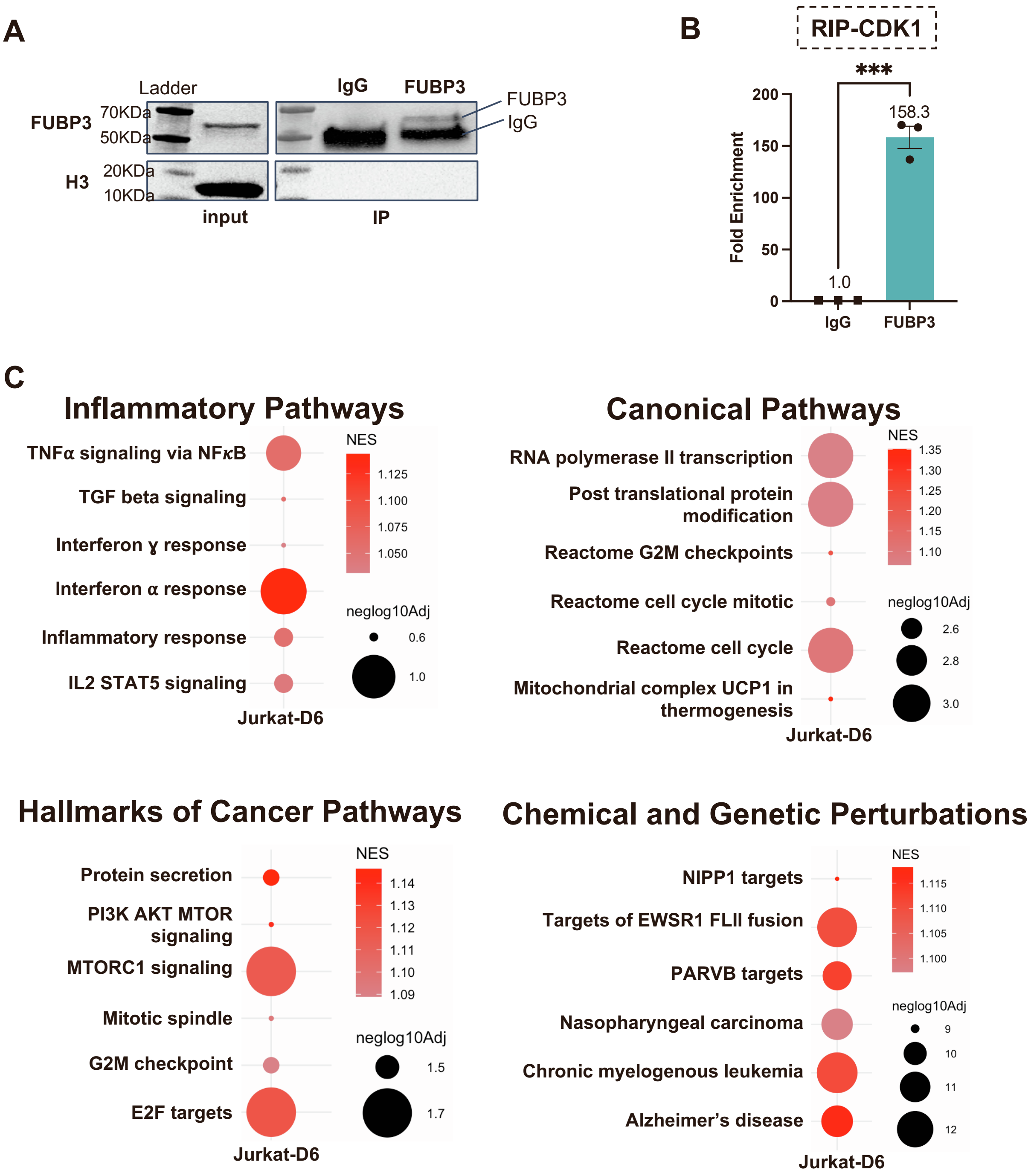

Figure S17. FUBP3 binds *TAR-RNA* in Jurkat-D6 cells. (A) Representative image of western blot depicting the pull-down of FUBP3 following RIP in Jurkat-D6 cells. (B) RIP validation against control IgG or FUBP3 subjected to qPCR to measure *CDK1* mRNA level in Jurkat-D6 cells. (C) Bubble plot of gene set enrichment analysis (GSEA) results from the RIP-seq data. NES: normalized enrichment score. ***, $p < 0.001$; as determined by student T-test. All data are reported as the mean ± SEM.

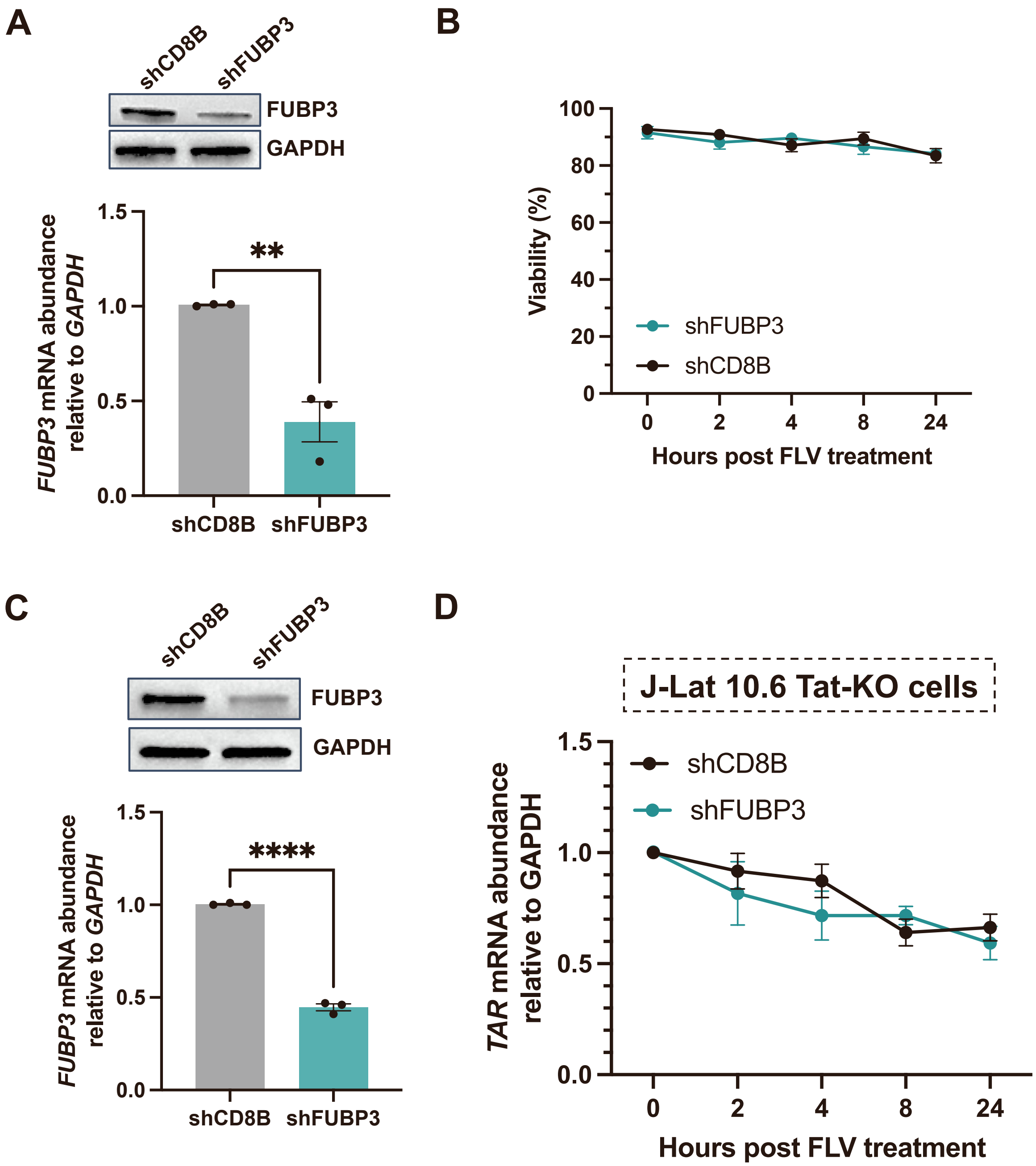

Figure S18. FUBP3 stabilizes *TAR-RNA* in J-Lat 10.6 cells Tat-dependently. (A) Knock-down efficiency for FUBP3 monitored by western blot (top) and RT-qPCR (bottom) in J-Lat 10.6 cells. (B) Cell viability overtime post treatment with flavopiridol [100 nM] monitored by Trypan blue staining. (C) Knock-down efficiency for FUBP3 monitored by western blot (top) and RT-qPCR (bottom) in J-Lat 10.6 Tat-KO cells. (D) *TAR-RNA* degradation with FUBP3 or CD8B deletion. J-Lat 10.6 Tat-KO cells were depleted by shRNAmir, followed by TNF-α [10 ng/mL, 6h] + TSA [1.5 μM, 6h] stimulation and flavopiridol treatment [100 nM] to inhibit transcription. *TAR* mRNA was quantified by RT-qPCR overtime. **, $p < 0.01$; ****, $p < 0.0001$; as determined by student T-test. All data are reported as the mean ± SEM.

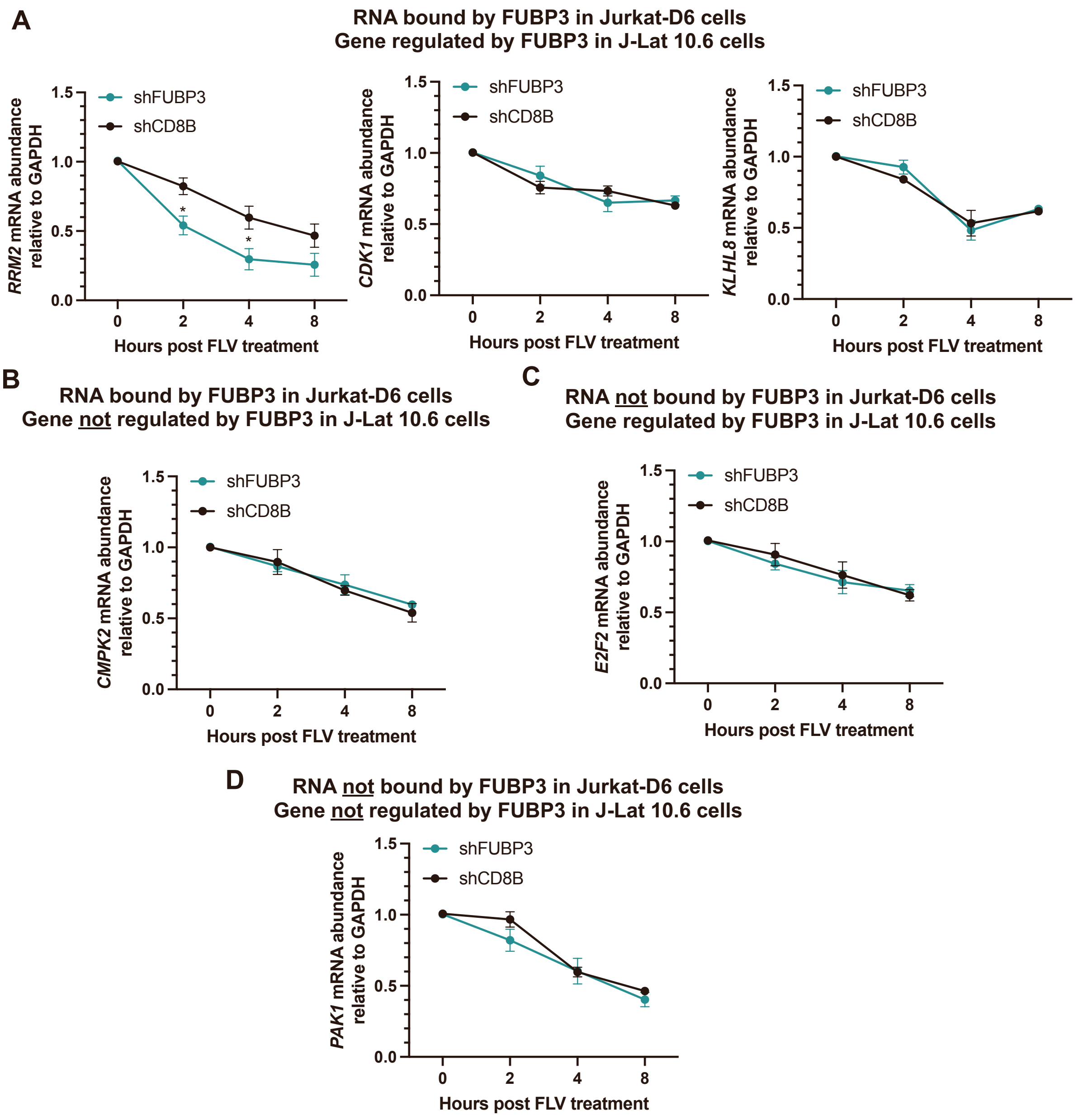

Figure S19. FUBP3 stabilizes *RRM2*-RNA in J-Lat 10.6 cells. (A)-(D): mRNA degradation with FUBP3 or CD8B deletion. J-Lat 10.6 cells were depleted by shRNAmir, followed by flavopiridol [100 nM] treatment to inhibit transcription. mRNA was quantified by RT-qPCR overtime. (A) Selection of 3 genes (*RRM2*, *CDK1* and *KLHL8*) significantly downregulated upon FUBP3 depletion in J-Lat 10.6 cells and with the RNA bound by FUBP3 by RIP-Seq in Jurkat-D6 cells. (B) Selection of 1 gene (*CMPK2*) not significantly affected upon FUBP3 depletion in J-Lat 10.6 cells but with the RNA bound by FUBP3 by RIP-Seq in Jurkat-D6 cells. (C) Selection of 1 gene (*E2F2*) significantly downregulated upon FUBP3 depletion in J-Lat 10.6 cells but without the RNA bound by FUBP3 by RIP-Seq in Jurkat-D6 cells. (D) Selection of 1 gene (*PAK1*) neither significantly affected upon FUBP3 depletion in J-Lat 10.6 cells nor with the RNA bound by FUBP3 by RIP-Seq in Jurkat-D6 cells. *, $p < 0.05$; as determined by student T-test or by two-way ANOVA with multiple comparisons. All data are reported as the mean ± SEM.

Table S1: Oligos used for cloning and pull-down		
**Name**	**Sequence**	**Notes**
shCD8B in pMKO-puro	GGCATCTACTTCTGCATGATC	Target of shRNA/shRNAmir
shCCNT1 in pMKO-puro	TGCATCGATTCTACATGATTC	
shFUBP1 in pMKO-puro	CTGCAGGTGCATCAACTACAA	
shFUBP2 in pMKO-puro	AAGAGCAAAGCAGAACTTATA	
shFUBP3 in pMKO-puro	AATGCTTGAAA TGTCTAACTA	
shCD19 in LMPd-Am	CCCCGCTTAAACCCTTCTTA	
shFUBP3 in LMPd-Am	AGGATTCAGTTTAAACCAG	
Δ1-77-147-R	CGTCCTTTGATGTACCAAGGCC	FUBP3 cloning, deletion of the KH domains
Δ1-77-147-F	GTACATCAAAGGACGTGTCGAAATGGACCTGGCTTTC	
Δ2-162-228-R	GTTGCTGTCTATGTCATTATGAAAGCC	
Δ2-162-228-F	ATAATGACATAGACAGCAACCTAGAGATTATCCGAG AAAAAGACCAAG	
Δ3-253-317-R	TCCCATTCGAGAGTTGAAATCGC	
Δ3-253-317-F	ATTTCAACTCTCGAATGGGAAGCGAGCTGATTCTTA CAGCCC	
Δ4-354-421-R	TGGTCCCGCCAACTTTCTCATCGCCACCAGGGGCT CCCACGCT	
Δ4-354-421-F	GATGAGAAAGTTGGCGGGACCA	
TAR-Bio3’ (RNA)	GGGUCUCUCUGGUUAGACCAGAUCUGAGCCUGGGAGC UCUCUGGCUAACUAGGGAACCC	RNA/DNA pulldown
TAR-Bio3’ (DNA)	GGGTCTCTCTGGTTAGACCAGATCTGAGCCTGGGAGC TCTCTGGCTAACTAGGGAACCC	
TAR-Del-Bio3’ (RNA)	GGGUCUCUCUGGUUAGACCAGCCUGGGAGCUGGCUAA CUAGGGAACCC	
TAR-Mut-Bio3’ (RNA)	GGGUCUCUCUGGUUAGACCAGGAAAGAGCCUGGGAGC UCUCUGGCUAACUAGGGAACCC	

Table S2: Primers used for qPCR and PCR		
**Name**	**Sequence**	**Notes**
FUBP1-F	GCAAGGCAGGATTAGTCATTGGA	mRNA analysis
FUBP1-R	CTCTAACACCATTTCCTTGGCTTG	
FUBP2-F	CGACAGTGAATAACAGCACTCCT	
FUBP2-R	GGACCCTGTACTCTTCTGTCATTG	
FUBP3-F	GTAACCAGTTAGGGGCCTTGGTA	
FUBP3-R	ACACAGGGCCTCTCTGGAATC	
CCNT1-F	ACAACAAACGGTGGTA TTTCACT	
CCNT1-R	CCTGCTGGCGA TAAGAAAGTT	
RRM2-F	TGGTCGACAAGGAGAACACG	
RRM2-R	CCAGGCATCAGTCCTCGTTT	
KLHL8-F	CGCAACAGTGATGGGCAAAA	
KLHL8-R	CAGCTCTGCAGTGAGACACA	
CCR4-F	GGAGGAAGGCTTACACCCAC	
CCR4-R	CCTCAAATAAATGTATTCTTGGCCT	
IL7R-F	CTCTGTCGCTCTGTTGGTCAT	
IL7R-R	CCAGGCATGTGAGGGATGAA	
E2F2-F	CAACATCCAGTGGGTAGGCA	
E2F2-R	GGCAATCACTGTCTGCTCCT	
CD2-F	TGTCAGCTGTCCAGAGAAAGG	
CD2-R	GGACGATGACTAGGTGCCTG	
Gag-pol-F	TTCTTCAGAGCAGACCAGAGC	
Gag-pol-R	GTTGCCAAAGAGTGATCTGA	
TAR-F	GGTTAGACCAGATCTGAGCCTGG	
TAR-R	CAACAGACGGGCACACACTACT	
Tat-Rev-F	CTTAGGCATCTCCTATGGCAGGAA	
Tat-Rev-R	GGATCTGTCTCTGTCTCTCTCTCCACC	
Tat-F	ATGGAGCCAGTAGATCCTAG	
Tat-R	GGGTTGCTTTGATAGAGAAACTTG	
GAPDH-F	CAACAGCCTCAAGATCATCAGCA	
GAPDH-R	AGGGATGACCTTGCCCACAGCCTTGG	
RPL13A-F	GCCCTACGACAAGAAAAAGCG	
RPL13A-R	TACTTCCAGCCAACCTCGTGA	
Alu-F	CCTGTGTCAGCTGCTGCTTG	ALU-PCR
Alu-Gag-R	TCCCAGCTACTGGGGAGGCTGAGG	
MSS-F	GGCTAACTAGGGAACCCACTG	
MSS-R	CTGCTAGAGATTTTCCACACTGAC	

Table S3: Primers used for ChIP-qPCR		
**Name**	**Sequence**	**Notes**
123-F	CCCTGATTGGCAGAACTACACAC	qPCR for ChIP
123-R	TCTACCTTATCTGGCTCAACTGGT	
403-F	GGGACTTTCCGCTGGGGAC	
403-R	CCCAGTACAGGCAAAAAGCAGC	
550-F	TCTCTGGCTAACTAGGGAACC	
550-R	AAAGGGTCTGAGGGA TCTCTAG	
610-F	AGTGTGTGCCCGTCTGTTGT	
610-R	TTCGCTTTCAAGTCCCTGTT	
800-F	GCGACTGGTGAGTACGCCAA	
800-R	CCCCTGGCCTTAACCGAATTT	
1121-F	GCAGTCCTCTATTGTGTGCATCAA	
1121-R	CTGGAGGTTCTGCACTATAGGGTA	
2940-F	GTACTGGATGTGGGCGATGCATA	
2940-R	CCATCCCTGTGGAAGCACATTG	
4745-F	CTACAATCCCCAAAGTCAAGGAGT	
4745-R	GTCTACTATTCTTTCCCCTGCACT	
7048-F	CAACTCAACTGCTGTTAAATGGCA	
7048-R	CTGGTCCCCTATGGATACGGATA	
9051-F	CCAGTCACACCTCAGGTACCTT	
9051-R	GGAAGTAGCCTTGTGTGTGGTA	
GAPDH-ORF-F	CCTCACGTATTCCCCCAGGTTTA	
GAPDH-ORF-R	AGCCACACCATCCTAGTTGCCT	

References

1. Li, C., Mori, L.P., Lyu, S., Bronson, R., Getzler, A.J., Pipkin, M.E., and Valente, S.T. (2023). The chaperone protein p32 stabilizes HIV-1 Tat and strengthens the p-TEFb/RNAPII/TAR complex promoting HIV transcription elongation. Proc Natl Acad Sci U S A *120*, e2217476120.
